# Need for speed: An optimized gridding approach for spatially explicit disease simulations

**DOI:** 10.1371/journal.pcbi.1006086

**Published:** 2018-04-06

**Authors:** Stefan Sellman, Kimberly Tsao, Michael J. Tildesley, Peter Brommesson, Colleen T. Webb, Uno Wennergren, Matt J. Keeling, Tom Lindström

**Affiliations:** 1 Department of Physics, Chemistry and Biology, Division of Theoretical Biology, Linköping University, Linköping, Sweden; 2 Department of Biology, Colorado State University, Fort Collins, CO, United States of America; 3 Zeeman Institute (SBIDER), School of Life Sciences and Mathematics Institute, University of Warwick, Gibbet Hill Road, Coventry, CV4 7AL, UK; University of New South Wales, AUSTRALIA

## Abstract

Numerical models for simulating outbreaks of infectious diseases are powerful tools for informing surveillance and control strategy decisions. However, large-scale spatially explicit models can be limited by the amount of computational resources they require, which poses a problem when multiple scenarios need to be explored to provide policy recommendations. We introduce an easily implemented method that can reduce computation time in a standard Susceptible-Exposed-Infectious-Removed (SEIR) model without introducing any further approximations or truncations. It is based on a hierarchical infection process that operates on entire groups of spatially related nodes (cells in a grid) in order to efficiently filter out large volumes of susceptible nodes that would otherwise have required expensive calculations. After the filtering of the cells, only a subset of the nodes that were originally at risk are then evaluated for actual infection. The increase in efficiency is sensitive to the exact configuration of the grid, and we describe a simple method to find an estimate of the optimal configuration of a given landscape as well as a method to partition the landscape into a grid configuration. To investigate its efficiency, we compare the introduced methods to other algorithms and evaluate computation time, focusing on simulated outbreaks of foot-and-mouth disease (FMD) on the farm population of the USA, the UK and Sweden, as well as on three randomly generated populations with varying degree of clustering. The introduced method provided up to 500 times faster calculations than pairwise computation, and consistently performed as well or better than other available methods. This enables large scale, spatially explicit simulations such as for the entire continental USA without sacrificing realism or predictive power.

## Author summary

Numerical models for simulating the outbreak of infectious disease are powerful tools that can be used to inform policy decisions by simulating outbreaks and control actions. However, they rely on considerable computational power to explore all outcomes and scenarios of interest. Focusing on model types commonly used for livestock diseases, we here introduce novel algorithms for efficient computation, alongside techniques to optimize them based on simplifying assumptions. Through simulations of FMD outbreak in the US, the UK and Sweden, as well as in computer generated landscapes, we test how these methods perform under realistic conditions. We find that our optimization techniques works well, and when the introduced algorithms are implemented with these optimizations, computation time can be reduced by more than two orders of magnitude compared to pairwise calculations. We propose that the considered algorithms—which are straight forward to implement—will be useful for simulation of a wide range of diseases, and will promote the use of simulation models for policy recommendation.

## Introduction

Models of infectious diseases are powerful tools for studying outbreak dynamics. Mass action mixing models assume equal probability of infection among all individuals in a population and have provided important theoretical insights for epidemiology. However, the importance of deviations from this assumption is now largely recognized [1], and researchers are increasingly implementing stochastic simulation models that incorporate various levels of realism [2].

The effect of spatial heterogeneity can have a pronounced effect on outbreak dynamics [3] and is of particular concern when models are used to inform policy. Spatially explicit models can be used to identify geographical hotspots targeted for surveillance [4] or to compare control scenarios that are themselves spatially explicit (e.g. ring vaccination strategies or regional movement restrictions). Here we focus on livestock disease models, but emphasize that the proposed methods are very broadly applicable. Livestock models typically consider infections at the farm level [5], and since the farms have fixed spatial locations, spatially explicit models are appropriate. Distance dependent transmission is commonly modeled with a spatial kernel that describes how transmission risk varies with distance [5–7].

Large livestock disease outbreaks can have severe societal and economic implications, necessitating models that can simulate outbreaks at the national or even the continental scale [7–9]. With a large number of farms, computation time may be a limiting factor, particularly when stochastic simulation models are used to quantify uncertainty when comparing across multiple scenarios. In the absence of efficient algorithms to improve computational time, pairwise calculations must be implemented, whereby the distances between every pair of infectious and susceptible farms need to be calculated and the spatial, transmission kernel must be evaluated for those distances. When the spatial kernel is narrow compared to the spatial distribution of farms, there will be many farms that lie within the tail of the kernel where the risk of infection is low. Given this relatively low risk, it may be tempting to truncate the kernel at such large distances in order to dramatically reduce the computation time of the simulation. However, it is difficult to identify a spatial scale where such truncation does not influence the results and finding such a scale by trial and error may become a time consuming process. Rare long distance transmission, described by a fat tail of the spatial kernel, can have a pronounced effect on outbreak dynamics, sparking new infections in virgin areas of susceptible farms [5]. In addition, with two-dimensional space (and assuming homogeneous farm locations), the number of potential transmission events also grows linearly with distance. Thus, even if the likelihood of infection of individual farms is low at large distances, there are many farms that can be infected and the probability of a transmission event occurring is not necessarily small. We therefore argue that truncation should be avoided due to its inaccuracy and that effort should be made to create algorithms to model transmission that circumvent the excessive number of kernel evaluations required for pairwise evaluation while maintaining accuracy. Brand et al. [10] introduced one such method, denoted the fast spectral rate recalculation (FSR). This approach utilizes fast Fourier transformation (FFT) of the spatial kernel, and with only slight approximation of the transmission rates, it can speed up simulations by two order of magnitudes.

Keeling and Rohani [11] introduced another technique, hereafter denoted the conditional entry algorithm, where the point pattern landscape of farm locations is overlaid with a grid, and the simulation of infections is split up into two steps:

1. Does the infection possibly enter the grid cell?
2. If yes, which farms (if any) within the grid cell are actually infected?

Our study introduces a novel method, which we will refer to as the conditional subsample algorithm. It builds on the approach of Keeling and Rohani [11], and utilizes a similar gridding approach but with a different algorithm. Importantly, the conditional entry and conditional subsample methods do not approximate the epidemiological process (beyond the temporal discretization, which are typically implemented also in pairwise simulation) that is simulated; they merely speed up the computation by reducing the number of calculations, and hence preserve the accuracy of the simulation.

**Fig 1:**
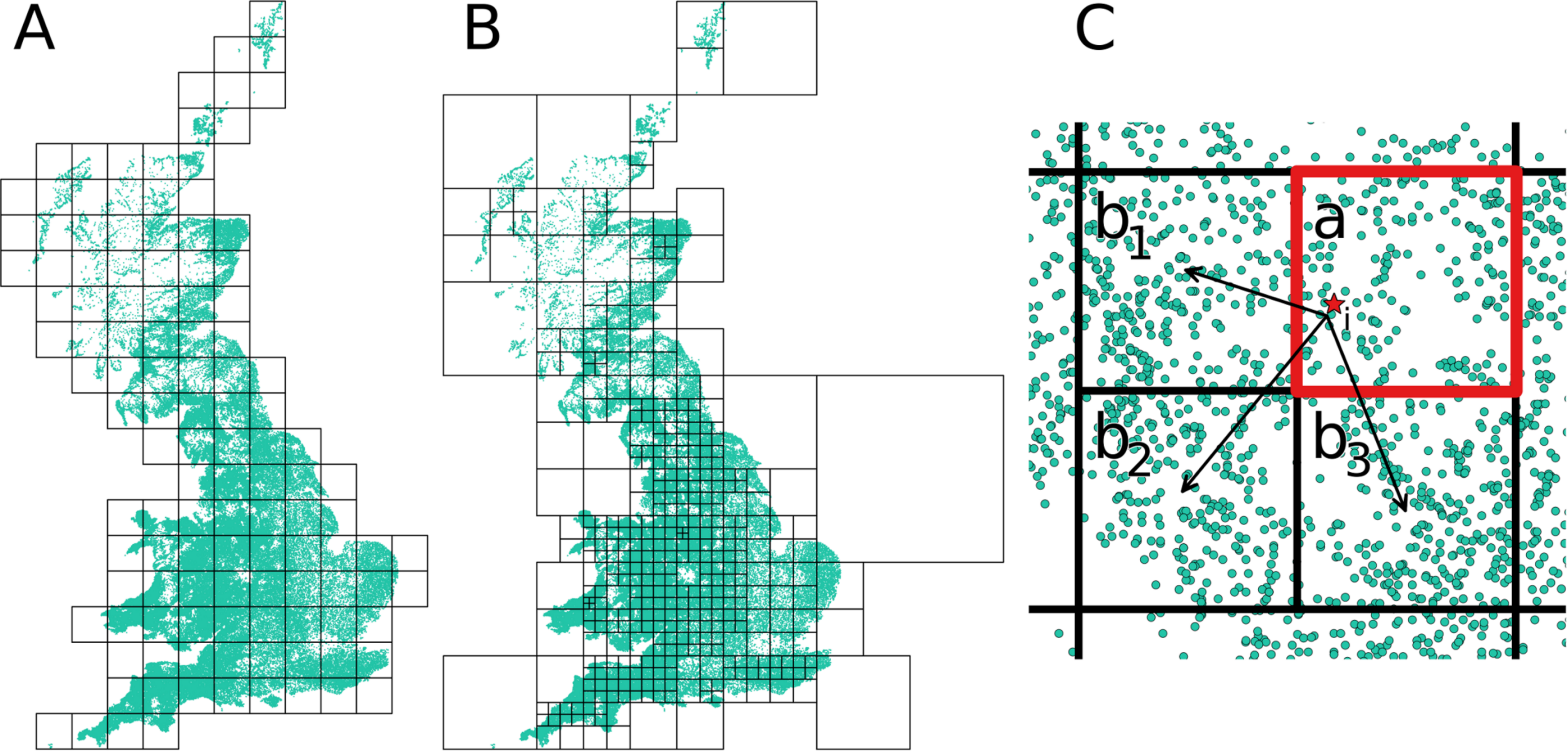
**Example of the regular (A) and the adaptive (B) grid construction methods applied to the UK farm population.** Panel (C) shows a schematic representation of the grid-based infection transmission process. From the infectious farm *i* (red star) in infectious cell *a*, spread of infection is initially evaluated at the level of entire susceptible grid cells (*b*_*1*_, *b*_*2*_, *b*_*3*_) and only occasionally evaluated for the individual susceptible nodes (green).

Implementing the conditional entry or conditional subsample method requires the specification and construction of a grid structure (Fig 1), which raises a central question: how many farms should each grid cell contain in order to facilitate fast computation? At both very large and very small cell sizes, both the conditional entry and conditional subsample algorithms require (at least) as many kernel evaluations as the pairwise algorithm. Thus, some intermediate grid size should optimize the speed of both algorithms.

This study has three aims. Firstly, we introduce a novel transmission algorithm for grid-based calculations: the conditional subsample algorithm. Secondly, we propose an optimal grid size estimation method for determining a grid size configuration for the conditional subsample and conditional entry algorithms that ensures fast computation, and evaluate its performance. Thirdly, we investigate how this algorithm compares to simulations based on both the pairwise algorithm, the conditional entry algorithm presented by Keeling and Rohani [11], and the FSR algorithm introduced by Brand et al. [10]. To address these aims, we simulate outbreaks on computer-generated farm locations with different levels of spatial clustering, as well as empirical farm populations from the USA, the UK and Sweden. These spatially heterogeneous farm distributions offers more realistic challenges to the proposed methods than would homogeneous distributions.

## Method

The methods of this study has several steps. We start by outlining the epidemic model, and present three primary transmission algorithms used for simulations: pairwise, conditional entry, and the novel conditional subsample algorithm. The two latter rely on similar gridding approaches, and we propose a straightforward optimal grid size estimation method that can be used for two of these algorithms. This estimation process formulates an equation that approximates the expected number of kernel evaluations for a particular average grid cell size, *θ* (expressed as farms per cell), and subsequently find the value of *θ* for which the fewest number of kernel calls are predicted. We denote the true unknown optimum θ* and the estimated equivalent ${\hat{\theta }}^{*}$. The estimation of ${\hat{\theta }}^{*}$ was based on the simplified assumptions of homogenous spatial distribution of farms and animals. We therefore simulated outbreaks of FMD with a set of *θ*, including ${\hat{\theta }}^{*}$, to explore how well the prediction holds when these assumptions are relaxed. The value of *θ* that performs best in the simulations is denoted by $\theta_{\mathrm{sim}}^{*}$ and will be equal to ${\hat{\theta }}^{*}$ if the proposed method performs well. The grids were constructed with two different methods that either retains equal cell sizes, or equal number of farms in each cell (Fig 1). Table 1 provides an overview of the different methods and how they fit together.

**Table 1:** Methods and epidemic model considered in the study and how they relate to each other.

Method/model	Description
Epidemic model	The epidemic process that is simulated.
Transmission algorithms	Algorithms used to simulate disease transmission of the epidemic model. Three algorithms are considered: pairwise, conditional entry (CE), and conditional subsample (CS).
Estimation of optimal grid size	Method used to an estimate of the optimal average grid size, ${\hat{\theta }}^{*}$, for the CE and CS transmission algorithms.
Grid construction method	Methods used to overlay a spatial grid of square cells on top of a landscape, assigning each farm to a cell. Two construction methods are implemented, retaining either equal cell sizes (regular grid construction, Fig 1A), or equal number of farms in each cell (adaptive grid construction, Fig 1B).

To further evaluate the performance of the two gridding based algorithms, we compared them to an algorithm proposed by Brand et al. [10]. We here simulated FMD outbreaks with kernel functions implemented in that study, and applied our introduced gridding optimization approach to the CE and CS algorithms. Thereby, we challenged the gridding based algorithms and the associated optimizations outside of the context used to initially explore their efficiency.

### Epidemic model

We use a spatially explicit kernel model based on an approach developed for the FMD outbreak in the UK in 2001 [5]. This is a versatile and common modeling approach that has also been used to model FMD in other countries such as the Netherlands [12] and Japan [6]; as well as other diseases, such as avian influenza in the USA [13] and bluetongue virus in the UK [14]. Our formulation is a standard farm level Susceptible-Exposed-Infectious-Removed (SEIR) model with discrete daily time steps and a daily rate of infection, *R*_*ij*_, between infectious farm *i* and susceptible farm *j*. This rate depends on the kernel *K*, which is a function of the Euclidean distance between *i* and *j* (*d*_*ij*_), as well as the transmissibility of *i* (*T*_*i*_) and the susceptibility of *j* (*S*_*j*_). The daily infection rate is given by

$$R_{ij}=T_{i}S_{j}K\left(d_{ij}\right).$$ (1)

The probability of a susceptible farm *j* becoming infected by farm *i* within a given span of days is obtained by discretizing Eq (1)

$$p_{ij}=1-\exp\left(-S_{j}T_{i}K\left(d_{ij}\right)\delta t\right)$$ (2)

where *δt* is the time step. By setting *δt* to one, *p*_*ij*_ becomes the daily probability of transmission from *i* to *j*. Once a farm was infected, an incubation period of four days was assumed after which the farm’s entire animal population was considered infectious for a period of five days and at the end of the infectious period the farm was considered removed from the population.

Following Tildesley et al. [15], transmissibility and susceptibility of farms were modeled as a nonlinear function of the number of cattle and sheep present on the premises as

$$T_{i}=\phi_{\mathrm{cattle}}z_{\mathrm{cattle},i}^{\tau_{\mathrm{cattle}}}+\phi_{\mathrm{sheep}}z_{\mathrm{sheep},i}^{\tau_{\mathrm{sheep}}}$$ (3)

$$S_{i}=\psi_{\mathrm{cattle}}z_{\mathrm{cattle},i}^{\sigma_{\mathrm{cattle}}}+\psi_{\mathrm{sheep}}z_{\mathrm{sheep},i}^{\sigma_{\mathrm{sheep}}},$$ (4)

where all parameters are species-specific constants, and *z* is the number of animals of the respective species on the individual farms. For simplicity, we initially used the set of parameter values for *τ*, *σ*, *Φ* and *Ψ* fitted for Cumbria, England in [15] for all simulations (see Table 2 for parameter values). These parameters resulted in outbreaks large enough to enable exploration of the efficiency of different grid configurations for the UK, but yielded small outbreak sizes when simulating infection across the other landscapes (S1 Table). We were also interested in investigating the performance of the algorithms for large outbreaks, and therefore multiplied the *ψ*_cattle_ estimated for UK (Cumbria) by 200 when simulating outbreaks in all other landscapes, which resulted in substantially larger epidemics. The animal populations for these other landscapes contained only cattle, thus obviating the sheep terms in Eqs (3) and (4).

**Table 2:** Infection kernel parameter values used in disease simulations.

Parameter			Reference
*φ*_sheep_	0.00083	Transmissibilty constant for sheep.	[15]
*φ*_cattle_	0.00082	Transm. constant for cattle.	.
*τ*_sheep_	0.49	Transm. exponent for sheep.	.
*τ*_cattle_	0.42	Transm. exponent for cattle.	.
*ψ*_sheep_	1.0	Susceptibility constant for sheep.	.
*ψ*_cattle_	5.7	Susc.constant for cattle.	.
*σ*_sheep_	0.20	Susc. exponent for sheep.	.
*σ*_cattle_	0.41	Susc. exponent for cattle.	.
*α*	0.089	Distance kernel normalization constant.	[7]
*β*	1600	Distance kernel scale parameter.	.
*γ*	4.6	Distance kernel shape parameter.	.

The local spread kernel, *K*, models the change in infection risk with distance between *i* and *j*, and includes all possible routes of infection except the shipment of animals between farms. For the purpose of comparing CE and CS and different optimized gridding schemes, we used the functional form

$$K(d_{ij})=\frac{\alpha }{1+{\left(\frac{d_{ij}}{\beta }\right)}^{\gamma }},$$ (5)

parameterized as in [7], (Table 2) and referred to as the *Buhnerkempe* kernel.

### Transmission algorithms

In this section, we consider three transmission algorithms: the pairwise (PW), the conditional entry (CE) and the conditional subsample (CS) algorithms. The CE algorithm has previously been published by Keeling and Rohani [11], whereas the CS algorithm is a novel approach. Example code for these three algorithms is provided as part of a C++ infection simulation model in the supplementary materials (S1 Appendix and S2 Appendix). The transmission algorithms were compared using simulation run times. However, since this is a measure that is both system dependent and also possibly influenced by other processes running simultaneously on the system we also determined the total number of calls made to the distance kernel function (Eq. 5) during a simulation. We considered this a measure of computational complexity and a proxy for relative computational time. Making a call to the kernel function is usually a relatively costly operation in itself and in a naïve implementation it will constitute most of the activity during a simulation as it takes place every time an infection probability is evaluated. For small populations of farms, it may be feasible to store the evaluated kernel values for each pair of farms in order to reduce the number of calculations, but for models on the individual farm level on national scales this approach easily becomes limited by memory availability. For comparison, representing the distances between all unique pairs as 64 bit double precision floating point numbers in a population of *n* nodes and making use of the fact that such a matrix is symmetrical to only store the upper or lower triangle requires approximately 0.373 GiB for n = 10^4^; 37.3 GiB for n = 10^5^ and 3725.3 GiB for n = 10^6^ (2691.5 GiB for n = 850000, which is roughly the size of the US farm population used in this study). Although supercomputer systems generally have memory per node in the ranges of 32 to 256 GiB, the availability of nodes in the higher range is usually limited and going above the limit of what a single node can handle necessitates a code that can handle shared memory between nodes, making a relatively simple problem significantly more complex. Also, even if it would be possible to run a simulation requiring a large amount of memory, lowering the memory requirement may allow multiple such simulations to run in parallel on the same system. A large number of independent replicates are generally needed, making numerical models for disease simulation prime examples of problems subject to embarrassingly simple parallelization. For the general case, this means that parallelization can be performed over the different replicates with no need for specialized code or extra overhead due to thread or process management, and that there is little to gain from more complex forms of parallelization.

Throughout the explanation of the algorithms we will refer to the farms as nodes. A cell containing an infectious node is referred to as *a*, while a cell with susceptible nodes is referred to as *b* (Fig 1C). The set of all cells that contain susceptible nodes is denoted **B**. An infectious node is referred to as *i*, a susceptible node as *j*. Sets of infectious and susceptible nodes are referred to as **I** and **J** respectively. These sets of nodes are sometimes subscripted with a cell to indicate a set of nodes within a cell. We use the notation of cardinality of the sets to refer to the number of elements in that set (e.g. |**J**_*b*_|, the number of susceptible nodes of cell b).

#### Pairwise algorithm

The pairwise algorithm is a straightforward approach for simulating transmission between nodes. The probability of infection is calculated directly for each infectious-susceptible pair in the population by Eq (2), and the probability of an infection occurring is evaluated as a Bernoulli process. While simple and easy to code, computational speed is a limiting issue for the pairwise algorithm. The method can be improved somewhat by pre-calculating distances or infection probabilities, but such an approach is instead very memory intensive for any but the smallest of populations, and here we provide the pairwise algorithm mostly for comparison, rather than a serious candidate for actual simulation work. The number of kernel function evaluations required per iteration ($N_{\mathrm{tot}}^{\mathrm{PW}}$) for the pairwise algorithm follows a bilinear relationship with the number of infectious, |**I**|, and susceptible, |**J**|, nodes, given by

$$N_{\mathrm{tot}}^{\mathrm{PW}}=\left|I\right|\left|J\right|.$$ (6)

**Pseudocode 1. Pairwise algorithm.** Probability *p*_*ij*_ as defined by Eq (2).

for each infectious node i {

    for each susceptible node j {

        R = uniform random number (0, 1)

        if R < p_ij_ {

            j is infected

        }

    }

}

The CE and CS algorithms utilize gridding approaches to partition the spatial landscape into grids. Although this partitioning of the landscape adds some overhead, it is small compared to the runtimes of the simulations. This is especially true if the nodes in the landscape are constant between replicates, since one grid can then be reused between runs. Unless the number of grid cells is exceptionally high, modern computers have enough memory to store the pre-calculated shortest distances or evaluated kernel values between all cell pairs to further minimize the number of calculations. The grid construction methods used to partition the landscapes are described after the transmission algorithms and the optimal grid cell size estimation algorithm have been outlined.

#### Conditional entry algorithm (CE)

With this method, the simulation of the infection process is separated into two steps:

1. Calculate the maximum possible probability of infection between the infectious node *i* in cell *a* and the most susceptible node within cell *b*, *υ*_*ib*_, using the minimum distance between the two cells. Based on this overestimated probability of infection, test if at least one susceptible node in cell *b* will be infected by *i*.
2. If yes, iterate over all susceptible nodes in *b* and test each individually to see which nodes (if any) actually become infected.

This approach has the benefit that if the outcome of step one is negative, only one evaluation of the kernel function will be required, thereby avoiding a large amount of computational operations. This is often the case at large distances, where the kernel predicts low transmission risk.

We denote the overestimated theoretical node-to-node level probability of step one as *υ*_*ib*_, obtained by assuming that the node in *b* with the highest susceptibility (${\hat{S}}_{b}$) is located at distance *d*_*ab*_, defined as the shortest possible distance between any two points within *a* and *b*, respectively

$$\upsilon_{ib}=1-e^{-T_{i}{\hat{S}}_{b}K\left(d_{ab}\right)}.$$ (7)

Given *υ*_*ib*_ and assuming that all susceptible nodes in *b* become infected with this probability, the probability of at least one of the susceptible nodes in *b* becoming infected is given by

$$\omega_{ib}=1-{\left(1-\upsilon_{ib}\right)}^{\left|J_{b}\right|}.$$ (8)

If a random draw *r*_*ib*_*~U([0*,*1)) > ω*_*ib*_, no further calculations are necessary to evaluate infections for nodes in *b* for the current infectious node *i* as not even one node became infected even with an overestimated infection probability. However, if *r*_*ib*_ *< ω*_*ib*_, there is a *possibility* of infection of each node in cell *b*, as evaluated in step two of the algorithm. Here, exact infection probability is evaluated for the nodes in *b*, based on actual distances and susceptibilities. However, the probability of infection needs to account for conditioning on the probability *ω*_*ib*_ of step one, that at least one hypothetical node at distance *d*_*ab*_ with susceptibility ${\hat{S}}_{b}$ must become infected. This is achieved by iterating over all susceptible nodes *j* in *b*, **J**_b_, and for each iteration calculate the probability of at least one of the remaining |**J**_*b*_|*-j* hypothetical nodes becoming infected, *χ*_*j*_:

$$\chi_{j}=1-{\left(1-\upsilon_{ib}\right)}^{\left|J_{b}\right|-j}.$$ (9)

The probability of *j* becoming infected at *d*_*ab*_ with susceptibility ${\hat{S}}_{b}$ conditional on *χ*_*j*_ is evaluated by comparing another random draw *r*_*j*_*~U([0*,*1])* to *υ*_*ib*_ */ χ*_*j*_. This ensures that the condition imposed in step one, that at least one node gets infected given the overestimated infection probability, will be fulfilled when *j =* |**J**_*b*_|*-1*, since at that point *χ*_*j*_ *= υ*_*ib*_. For every *j* where *r*_*j*_ *< υ*_*ib*_ */ χ*_*j*_, the node would have become infected if it was at *d*_*ab*_ and had susceptibility ${\hat{S}}_{b}$, so there is now also a risk of actual infection taking place. Furthermore, the first time this occurs, the condition from step one is fulfilled and *χ*_*j*_ for the subsequent |**J**_*b*_|*-j* iterations is set to 1. The probability of actual infection is *p*_*ij*_, given by Eq (2), conditional on *χ*_*j*_ such, that *j* becomes infected if *r*_*j*_ *< p*_*ij*_ */ χ*_*j*_. The same random number is reused in this step since the first test is essentially checking if infection can occur given the known upper bound of the probability (*υ*_*ib*_ */ χ*_*j*_), and then only calculating *p*_*ij*_ */ χ*_*j*_ if necessary. For within-cell infections (when *a = b*) the pairwise algorithm was used. For a formal proof of the exactness of this method, see the supplementary material (S3 Appendix).

**Pseudocode 2. Conditional entry algorithm.** Probabilities *p*_*ij*_, *υ*_*ib*_ and *ω*_*ib*_ as defined by Eqs (2), (7) and (8) respectively.

for each infectious node i {

    for each cell b {

        if i is not in b {

            R_1_ = uniform random number (0, 1)

            if R_1_ < ω_ib_ {

                N = set of susceptibles in b

                n = size of N

                over-estimated infection has occurred = false

                for j from j = 0 to j<n {

                    if over-estimated infection has occurred {

                        q = 1

                    } else {

                        q = 1 –(1 - υ_ib_)^n-j^

                    }

                    R_2_ = uniform random number (0, 1)

                    if R_2_ < υ_ib_ / q {

                        over-estimated infection has occurred = true

                        if R_2_ < p_ij_ / q {

                            N[j] is infected

                        }

                }

            }

        }

    } else {

        pairwise algorithm

        }

    }

}

#### Conditional subsample algorithm (CS)

Here, we introduce a novel transmission algorithm that shares several features with the CE algorithm. However, instead of either rejecting an entire cell or iterating through all the susceptible nodes of the cell, it selects a sub-sample of the cell’s population of susceptible nodes to be considered for infection. Similar to the CE method, it utilizes an overestimated probability of infection, *ω*_*ab*_, describing an upper boundary for the probability of infection spreading from any of the infectious nodes in cell *a* to a susceptible node in cell *b* in one time step (in contrast to spreading from one single infectious node *i* in *a* to any of the susceptible nodes *j* in *b* as in the CE method). We first define the upper boundary for the probability of one infectious node *i* in cell *a* infecting one susceptible node *j* in cell *b* based on over-estimated transmission parameters. This is similar to Eq (7) but not identical because we use the maximum transmissibility of any node in cell *a*, ${\hat{T}}_{a}$, in addition to the maximum susceptibility of any node in cell b, ${\hat{S}}_{b}$:

$$P({\hat{i}}_{a}\to {\hat{j}}_{b})=\upsilon_{ab}=1-e^{-{\hat{T}}_{a}{\hat{S}}_{b}K\left(d_{ab}\right)}$$ (10)

Using only over-estimated transmission parameters means that *υ*_*ab*_ will be constant for a given combination of *a* and *b*, since the maximum transmission parameters ${\hat{T}}_{a}$ and ${\hat{S}}_{b}$, as well as the distance that the calculation is based on remain unchanged throughout a simulation. Therefore, *υ*_*ab*_ can be pre-calculated for all cell pairs to improve performance. Now we define the probability that at least one of the infectious nodes in cell *a* infects one node in *b* given ${\hat{T}}_{a}$ and ${\hat{S}}_{b}$ as

$$\omega_{ab}=P(\hat{a}\to {\hat{j}}_{b})=1-{\left(1-\upsilon_{ab}\right)}^{\left|I_{a}\right|}.$$ (11)

Based on *ω*_*ab*_, the CS method simulates transmission by the following steps:

1. For each cell *a* containing at least one infectious node, iterate over all other cells *b* and generate a random number *n*_*b*_ from a binomial distribution with number of trials |***J***_*b*_| and probability *ω*_*ab*_. This gives the number of nodes that can potentially become infected given the upper boundary to the probability of infection.
2. Randomly select a subset of *n*_*b*_ nodes from *b*, each with equal probability of being selected.
3. For each of the nodes *j* in the selected subset of nodes, calculate the cumulative true probability of the event that *j* is infected by one or more of the infectious nodes in *a* as
  $$p_{aj}=P(a\to j_{b})=1-\prod_{i\in I_{a}}\left(1-p_{ij}\right)$$ (12)
  where *p*_*ij*_ is the true probability of infection spreading from *i* to *j* as given by Eq (2). Evaluate if infection actually takes place using the true probability conditional on the upper boundary of the probability used in step 1.

We treat the infection of node *j* by cell *a* as conditional on the event that it would be infected using the over-estimated transmission parameters,

$$P(\hat{a}\to {\hat{j}}_{b})P(a\to j_{b}|\hat{a}\to {\hat{j}}_{b})=P(\hat{a}\to {\hat{j}}_{b})\frac{P(a\to j_{b})}{P(\hat{a}\to {\hat{j}}_{b})}.$$ (13)

In practice this becomes two separate Bernoulli trials with probability $P(\hat{a}\to {\hat{j}}_{b})$ and $P(a\to j_{b})/P(\hat{a}\to {\hat{j}}_{b})$ respectively. Since the first of these two trials have equal probability regardless of which susceptible node *j* is being considered, their combined number of successes, *n*_*b*_, can be expressed as a binomial random variate. Because $P(\hat{a}\to {\hat{j}}_{b})$ is identical for all nodes *j*, all nodes have equal probability to pass the first Bernoulli trial where infection based on over-estimated transmission parameters is evaluated. Therefore, sample of *n*_*b*_ nodes from the susceptible population of cell *b*, where each susceptible node have equal probability of being selected yields the exact set of nodes to be tested for actual infection with probability $P(a\to j_{b})/P(\hat{a}\to {\hat{j}}_{b})$. Thereby, the actual probability of infection *p*_*aj*_ only needs to be calculated for a subsample of susceptible nodes each time step.

We once again stress that the CS algorithm is not an approximation of the pairwise algorithm; it reduces the number of times that the true infection probability *p*_*ij*_ needs to be calculated by using a single step to filter out nodes that would not have become infected even with the overestimated probability *ω*_*ab*_. Even though the operation of generating a binomially distributed random variable can be considered relatively costly, this extra complexity is small in relation to the number of evaluations of the kernel function that is avoided. A way to work around the computationally costly construction of binomial random generators each time a new *n*_*b*_ is drawn is to set up a number of generators for all combinations of range of fixed probabilities ***P***_CS_ and all possible values of n between 1 and the largest number of nodes within any cell given the grid in use. To generate *n*_*b*_ from this set of binomial generators one simply rounds *ω*_*ab*_ up to the closest fixed p in ***P***_CS_ this makes the over estimation of the actual infection probability even more crude giving a slightly larger *n*_*b*_ on average, but the time saved from not having to set up the generators will likely be much larger than the extra tests necessary. For the simulations in this work we used the set ***P***_CS_ *= {1*.*0*, *0*.*9*, *0*.*8*, *0*.*7*, *0*.*6*, *0*.*5*, *0*.*4*, *0*.*3*, *0*.*2*, *0*.*1*, *1*.*0e*^*-2*^, *1*.*0e*^*-3*^, *1*.*0e*^*-4*^, *1*.*0e*^*-5*^, *1*.*0e*^*-6*^, *1*.*0e*^*-7*^, *1*.*0e*^*-8*^, *5*.*0e*^*-9*^*}*. We point out that this approach simply makes the over-estimation of *ω*_*ab*_ slightly larger and does not introduce any approximation of the true probabilities of nodes becoming infected. The implementation of the binomial random number generator used will of course still impact the absolute speed of the algorithm and care should be taken to ensure that the most naïve implementations are avoided.

For within-cell infections (when *a = b*) the pairwise algorithm was used.

**Pseudocode 3. Conditional subsample algorithm.** Probabilities *p*_*ij*_, and *ω*_*ab*_ as defined by Eqs (2) and (11) respectively.

for each cell with infectious nodes a {

    for each cell with susceptible nodes b {

        if a is not b {

            N_b_ = set of susceptibles in b

            n_b_ = size of N_b_

            n_sample_ = binomial random variate(n_b_, ω_ab_)

            if(n_sample_ > 0) {

                N_a_ = set of infectious in a

                N_sample_ = random subsample of size n_b_ from N_b_

                for each j in N_sample_ {

                    p_aj_ = 1.0

                    for each i in N_a_ {

                        p_aj_ = p_aj_ * (1.0—p_ij_)

                    }

                    R = uniform random number (0, 1)

                    if R < (1 –p_aj_) / ω_ab_ {

                        N_sample_[j] is infected

                    }

                }

            }

        } else {

            pairwise algorithm

        }

    }

}

### Estimation of optimal grid cell size

The cell size (number of nodes contained in each cell) will have a large impact on the efficacy of both the CE and CS transmission algorithms. Conceptually, it is easy to see that either too small or too large cells will render the methods inefficient. At one extreme end, a single cell covering the entire landscape would equate the method to the pairwise algorithm. On the other hand, if each cell is so small that it only contains a single farm, the kernel similarly needs to be evaluated for every farm and iteration, plus an additional evaluation when a cell is entered. Clearly, some intermediate cell size is optimal.

In theory, the best cell configuration is one where the difference between the upper bound of the probability of infection (*υ*_*ib*_ or *ω*_*ab*_) and the probability of infection itself is as small as possible because this will lead to fewer ‘false positives’ (times where the outcome of step 1 is true for the CE algorithm, or a larger than necessary binomial sample for the CS algorithm). The amount of overestimation of *υ*_*ib*_ and *ω*_*ab*_ can be reduced by using small cells with few nodes in each since that will minimize the difference between *d*_*ab*_ (the cell-to-cell distance) and *d*_*ij*_ (the actual distance between the infectious node *i* and the susceptible nodes *j*). Furthermore, having a cell in which the distribution of the nodes’ susceptibility, *S*_*j*_, is as homogenous as possible also contributes to a smaller amount of overestimation. At the same time, the more small cells there are, the closer the behavior of the transmission algorithm will be to that of the pairwise algorithm in that there will be a larger amount of operations required just to evaluate part one of respective algorithm. Finding a grid configuration with an optimal balance between these factors for a landscape with a spatially heterogeneous node distribution is not a trivial problem. Also, any perfect solution will be dependent on where in the landscape the infectious node is, further complicating the task.

We propose a straightforward method to quickly find an approximation of the optimal average grid cell size, ${\hat{\theta }}^{*}$, for a given landscape. The method relies on a set of simplifying assumptions, where all farms are assumed to have equal susceptibility and transmissibility, and distributed uniformly in a quadratic landscape. Also, we only estimate ${\hat{\theta }}^{*}$ for the initial phase of the outbreak, where only one node is considered infectious and all other nodes are susceptible. Note, however, that we challenge these assumptions below when we evaluate the performance of the gridding methods. The method to identify an optimal grid size is:

1. Starting out with the landscape of interest, determine the longest side *x* of the surface delimited by the outer bounds of the node population and consider a square of area *x*^*2*^.
2. Overlay this square with a grid consisting of *κ*^*2*^ uniformly sized square cells and assign the nodes to the cells. This gives an average number of nodes per cell, $\theta =n{\hat{\kappa }}^{-2}$.
3. For each grid cell calculate the expected number of kernel function calls that would be required to simulate infection spreading from a single infectious node in this cell *a* to all cells (including itself) in the landscape with the current grid configuration. For these calculations let the number of nodes in each cell be the average number of nodes per cell, and let susceptibility and transmissibility be the same respective parameter value for all nodes. In our study we based these on the median of the number of animals (*z*_sheep,*i*_ and *z*_cattle,*i*_, Eqs (3) and (4)) within the farm populations, but stress that the approach could also be used when other demographic heterogeneities are considered.
4. Repeat step 2 and 3 with differently sized grid configurations.
5. Determine ${\hat{\theta }}^{*}$ as the value of *θ* yielding the fewest average number of expected kernel function calls per cell *a*.

This procedure was performed for 100 different grid configurations, each with total number of cells *κ*^*2*^ where *κ* ∈ {1,…,100}. For *κ* = 1, a single cell overlays the landscape, and 100 was chosen as an upper bound that we determined to yield a large enough span of grid configurations to be sufficiently certain that the optima for the landscapes used in this study were found. The sum of expected number of kernel calls required for each evaluated cell was calculated as

$$N_{tot}=\sum_{a\in C}\left(\sum_{\begin{array}{l} b\in C\\ b\neq a \end{array}}\left(N_{i,b}+1\right)+\left|J_{a}\right|\right),$$ (14)

where **C** is the set of all cells for the given configuration and *N*_*i*,*b*_ is the expected number of calls to the kernel function required to simulate infection spreading from one infectious node *i* in *a* to the nodes in *b* during one time step. For both the CE and CS transmission algorithms, there will always be one kernel call made for each cell that is checked in order to calculate the overestimated infection probability *υ*_*ib*_ or *ω*_*ab*_, as well as |**J**_*a*_|calls associated with the internal pairwise checks of the susceptible nodes within *a*. Note that since the distribution of animals is uniform over the nodes and the nodes are uniform within the landscape, the transmissibility parameters from Eq (7) and Eq (10), will have the same value ($T_{i}={\hat{T}}_{a}$) and the number of infectious nodes in *a* is one, so *υ*_*ib*_ = *ω*_*ab*_. After the initial overestimated probability *υ*_*ib*_ or *ω*_*ab*_ has been calculated, the expected number of kernel calls for a given pair of infectious node *i* (or cell *a*, it is the same in this context since there is only one infectious node in *a*) and grid cell *b* is

$$N_{i,b}=\upsilon_{ib}\left|J_{b}\right|.$$ (15)

This is simply the expected value of the binomial distribution given |**J**_*b*_| draws and probability *υ*_*ib*_ or *ω*_*ab*_. Eq (15) is intuitive for the CS algorithm because each node in the binomial sample, drawn from the population of susceptible nodes within a grid cell during the execution of the algorithm, will be evaluated exactly once. This adds one kernel call for each node in the sample. The proof is somewhat less apparent for the CE algorithm, requiring a more comprehensive derivation. This is provided in the supplementary material (S4 Appendix).

Due to *ω*_*ab*_ increasing with the number of infectious nodes within a cell it was expected that during actual simulations with the CS algorithm the probability *ω*_*ab*_ would be close to or equal to one more often than *υ*_*ib*_ would with the CE algorithm. This leads to the CS algorithm degenerating into the pairwise algorithm (when *ω*_*ab*_ = 1, *n*_*b*_ = |**J**_b_|) more often than the CE algorithm which will still only occasionally enter the susceptible cell *b*, even when a cell contains a lot of infectious nodes. In order to make a fair comparison between the methods we therefore also calculated ${\hat{\theta }}^{*}$ using the maximum number of animals on any farm in the landscape as well as the 75^th^ percentile of the number of animals on the farms for transmission parameters rather than the median, and evaluated both using the CS and CE algorithms. With these higher transmission parameters, the cells become somewhat smaller which offsets this effect for the CS algorithm. The results showed mostly minute differences in run times for simulations depending on summary statistic used, but for the CS method using maximum number of animals was clearly optimal for the UK (3.2 and 2.9 times faster than 75^th^ percentile and median, respectively). Based on this we chose to use maximum for all simulations with the CS algorithm and median for all simulations with the CE method.

### Grid construction

Two different methods were used to spatially divide the nodes of the landscapes into grids, each grid consisting of a set of square cells, **C**. The first such grid construction method, denoted regular grid construction, overlays the landscape with a set of uniformly sized square cells of predetermined spatial size (Fig 1A), generated by selecting a number *κ*, describing the square root of the total number of cells (or the number of cells along one dimension) desired, and simply constructing *κ*^*2*^ square cells with side = *l/κ*, where *l* is the longest side of the rectangle bounding the landscape.

Secondly, we used an adaptive grid construction approach with similarities to the quad-tree data structure common in computer science, where a spatially heterogeneous grid is constructed (Fig 1B), with the aim of having an equal number of nodes per cell. Starting with one large cell covering the entire landscape, we recursively divided cells (parents) into four smaller equal-sized cells (children), which in turn were divided into even smaller cells and so on. The process was continued for each cell as long as further subdivision satisfied the following condition:

$${\left(\log\left(\left|L_{a}\right|\right)-\log\left(\lambda \right)\right)}^{2}\geq \frac{\sum_{b\in B}\left({\left(\log\left(\left|L_{b}\right|\right)-\log\left(\lambda \right)\right)}^{2}\right)}{\left|B\right|}.$$ (16)

Here, **L**_*a*_ and **L**_**b**_ indicates the set of nodes within parent (*a*) and child (*b*) cells, respectively, **B** is the set of up to four child cells of *a* that contain nodes (depending on the spatial distribution of nodes inside the parent cell, some child cells can end up without nodes) and *λ* is the threshold number of nodes per cell. As such, the subdivision minimizes the squared difference on between log-number of nodes per cell and the specified log-*λ*.

At the end, all cells that did not contain any nodes were removed from the final grid for both the adaptive and the regular grid construction methods.

We applied the grid construction methods to farm landscapes that deviated from the underlying assumptions of the approximation in section *Estimation of optimal grid cell size* (uniform node distribution inside a square with equal susceptibility and transmissibility), using grids according to the optimized ${\hat{\theta }}^{*}$ as well as both finer and coarser grid configurations. We specified

$$\lambda_{i}=\eta^{i}{\hat{\theta }}^{*},i\in \left[-6,-5,\ldots ,6\right].$$ (17)

The constant *η* was set to 1.75 in order to give a large enough span of different grid configurations across all landscapes and the range of *i* was chosen as [6, 6] to yield a manageable number of grid configurations with a sufficient level of detail as well as equal amount smaller and larger cell sizes in addition to ${\hat{\theta }}^{*}$ itself. In order to make the regular grids somewhat comparable in size to the grids constructed with the adaptive method, the sets of values for *κ* were set to the integer values for which the average number of nodes per cell (*n/κ*^*2*^*)* was closest to the sets of values of *λ*.

For the landscape with uniform spatial distribution of nodes, the adaptive gridding approach generally resulted in configurations with cell sizes that deviated substantially from *λ*. Therefore, for this landscape, we used only the regular gridding method.

### Landscapes

The evaluation of the algorithms and the gridding schemes were performed on the empirical cattle and sheep farm population of the UK and the cattle farm populations of the 48 contiguous states of the USA (i.e. excluding Alaska and Hawaii) and Sweden, each with 177 855, 832 514 and 24 275 farms respectively; as well as on three generated landscapes with differing degrees of clustering. The generated landscapes were created with the method of [16] and each had one quarter of the number of farms of the USA data (208 129) and an area that was one tenth of that of the contiguous USA. This scaling of the random landscapes was found to provide consistently large outbreak sizes through high enough farm density, while keeping simulation runtimes with the pairwise method at a manageable level. The number of animals on the farms for the generated landscapes were sampled randomly from the USA farm size distribution. See S2 Table for clustering statistics of the landscapes.

The UK and Sweden keep detailed information about farms in central databases, including position and herd sizes. These data were made available for the study under confidentiality agreements and could be used when simulating outbreaks. There are however no equivalent data bases for the USA. Instead we used simulated demography data generated by the Farm Location and Agricultural Production Simulator (FLAPS). FLAPS simulates spatially-explicit farm locations and farm sizes, based on county level demography information from the National Agricultural Statistics Service (NASS), in combination with environmental features (e.g. topography and climate), and anthropogenic factors (e.g. roads and urban markets) [17]. While not a perfect representation of the geographical distribution and demography of the USA farm population, it offers the most realistic depiction available. As such, it is well suited for investigating the performance of the presented disease simulation algorithms.

The three randomized landscapes as well as the FLAPS realization for the USA farm population used in this study are available as supporting information (S1–S4 Datasets).

### Simulations

The usefulness of $\hat{\kappa }$ and ${\hat{\theta }}^{*}$ as indicators of the actual optimal grid size for the original landscape was tested by comparing them to a number of other configurations generated using the two different grid construction methods (regular and adaptive) through simulations. Each simulation was seeded with one random farm as the initial case and was run until the outbreak either died out or until 10,000 cumulative farms had become infected. The run time and total number of kernel operations depends heavily on the number of nodes infected over the course of the simulated outbreaks, making comparisons between replicates with different outbreak sizes difficult. To circumvent this problem, the number of kernel operations of the simulations were recorded at the end of the time step where 10, 100, 1,000 and 10,000 nodes became infected (we refer to these as outbreak stages).

To improve the speed of all simulations regardless of transmission algorithm the kernel was evaluated for every integer distance between 1 and the maximum possible distance within the given landscape (in meters) and the distances calculated during the simulations were rounded to nearest integer. This does not, however, mean that the number of kernel calls as a relative measure of complexity changes as it still indicates the number of operations made where the kernel is involved. Also, even though this can be seen as an approximation, we argue that the error introduced by this approach will be smaller than the error in a set of landscape coordinates at the one-meter level.

The simulations of the epidemic model using the grid construction methods and transmission algorithms, as well as the algorithm for finding ${\hat{\theta }}^{*}$, were all implemented in C++ (S1 Appendix, S2 Appendix). All simulations were run at a supercomputer cluster consisting of 2.2GHz 8-core Intel Xeon E5-2660 processors.

### Comparison to existing algorithms

The CE and CS algorithms were compared to the fast spectral rate recalculation method (FSR) presented by Brand et al. [10]. Simulations with the FSR method and the other algorithms was performed using the US farm population with parameters as described in the [10]

We compared the two methods using the distance kernel from the evaluation of the FSR method on the US population in [10] (referred to as *Brand* kernel; notation converted to match that already in this paper)

$$K_{\gamma }(d)=\alpha N_{\gamma }\beta {\left(\beta^{2}+d^{2}\right)}^{-\gamma /2}.$$ (18)

As closely as possible we tried to replicate the simulations in the original study and tested the methods with three different sets of parameters corresponding to three different shapes of the kernel (Table 3), *N*_*γ*_ being a normalization constant dependent on the choice of *γ*. In the comparison, the same node second closest to the center of Franklin County, Texas was seeded every replicate. The reason for choosing the node second closest to the center over the node closest to the center as in the original study, was that it had 19 animals in our data set as opposed to the central node which had only 1, increasing chances of outbreaks taking off. Estimation of optimum grid size (${\hat{\theta }}^{*}$) for the CS and CE method was performed as described under section *Estimation of optimal grid cell size* using the relevant kernel (Table 3). For these simulations, results were recorded at the time step when 10000 cumulative infected nodes was reached as well as at the point where the epidemic died out.

**Table 3:** Parameters for infection kernels used in the comparison to FSR method.

	Kernel			
	*Brand*	*Brand*	*Brand*	*Buhnerkempe*
*α*	0.12	0.12	0.12	0.089
*β*	10.0	12.73	20.0	1.6
*γ*	3.0	4.0	5.0	4.6
*N*_*γ*_	0.16	4.05	190.99	-
*φ*_*c*_	1.0	1.0	1.0	1140.0
*τ*_*c*_	0.2	0.2	0.2	0.00082
*ψ*_*s*_	1.0	1.0	1.0	0.41
*σ*_*c*_	0.2	0.2	0.2	0.42
${\hat{\theta }}^{*}$ (CS)	411	393	450	377
${\hat{\theta }}^{*}$ (CE)	925	925	925	308

*Brand* refers to the infection kernel used in [10] which was implemented using three different values for the shape parameter γ as in the original work. *Buhnerkempe* refers to the infection kernel of [7].

A comparison to the FSR method was also attempted for the kernel and set of parameters used for the other analyses presented earlier in this paper (Eq (5) and Table 3). However, the very local nature of this kernel caused problems with the FSR method as the kernel shape necessitates a very fine resolution for the grid on which the image of infection is calculated in order to accurately capture the behavior of the kernel. For such fine resolutions, the efficiency of the algorithm drastically diminishes and made simulations until the end of the epidemic too long to be feasible. Thus, for this kernel, only the results from reaching 10000 cumulative infected farms was recorded before the simulations were terminated.

Please note that the kernels used in the comparison with FSR are parameterized for a distance unit of kilometers rather than meters as for the other analyses presented.

## Results

**Fig 2:**
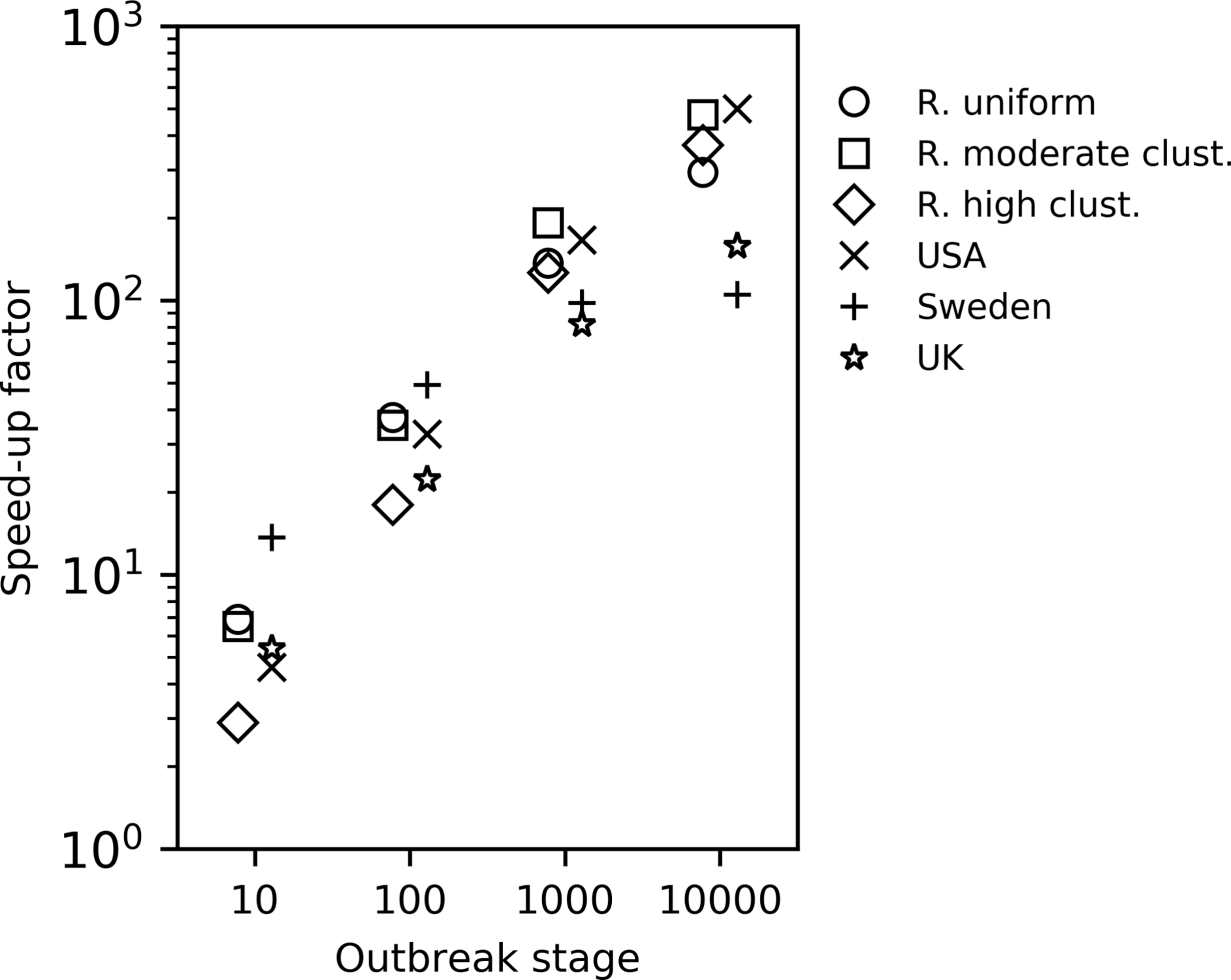
Speed-up of estimated optimal grid configuration with the conditional subsample algorithm compared to the pairwise algorithm. Comparison of simulations using the CS algorithm with ${\hat{\theta }}^{*}$ as cell-size threshold, with the pairwise algorithm for different outbreak stages. The y-axis indicate how many times faster (based on run time) the simulations using the CS algorithm were compared to the pairwise simulations.

**Fig 3:**
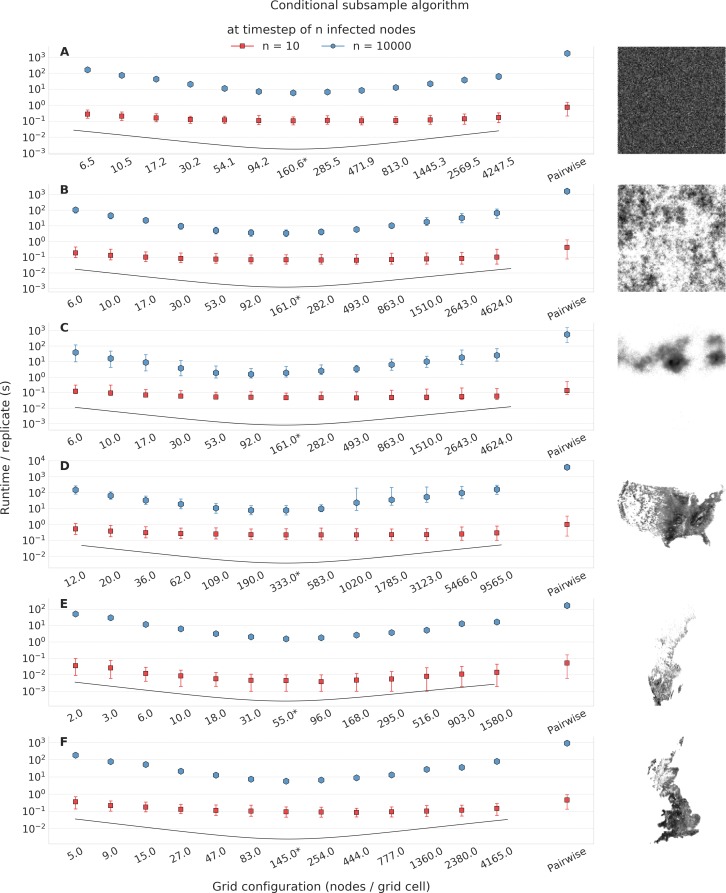
Results from outbreak simulations with the conditional subsample transmission algorithm. Average run time in seconds for each tested grid cell size up to and including the outbreak stages 10 and 10000 (* indicates estimated optimal grid cell size ${\hat{\theta }}^{*}$). The 5th and 95th percentiles are indicated by the ranges (main panels). Each combination of landscape and grid configuration using the CS algorithm, as well as simulations with the pairwise algorithm for comparison was simulated with 500 replicates. The landscapes were (panels A-F): random uniform, random moderate clustering, random high clustering, USA, Sweden, UK. The regular grid construction method was used for the uniform random landscape and the adaptive grid construction method was used for the other landscapes. The black line indicates a unitless relative expected efficacy of the different grid sizes as indicated by the grid optimum estimation method.

**Fig 4:**
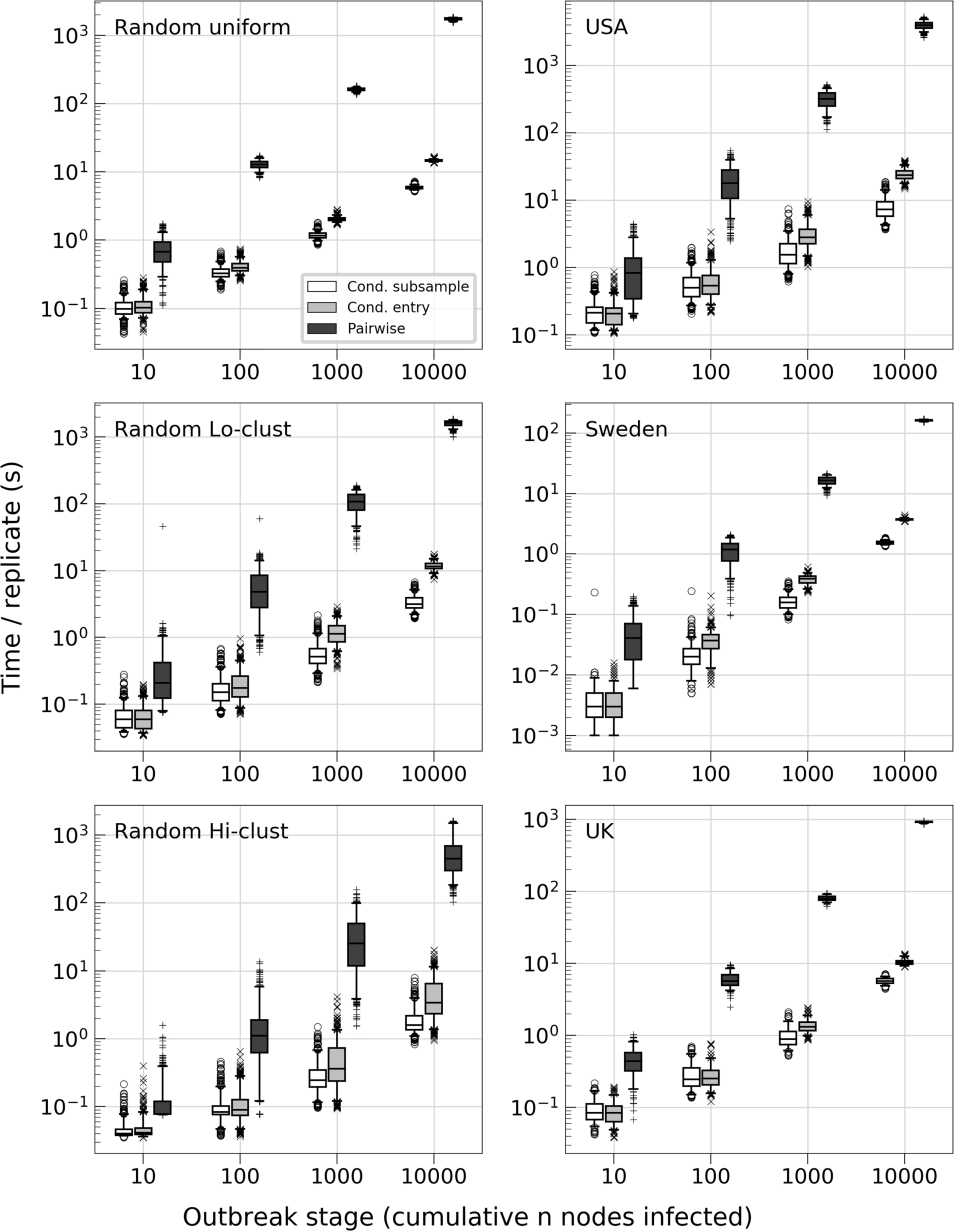
Transmission algorithm performance for estimated optimal grid size ${\hat{\theta }}^{*}$. Run time in seconds for simulations reaching 10, 100, 1000 and 10000 cumulative infected nodes. The grids were constructed using the adaptive gridding method using the estimated optimal grid size ${\hat{\theta }}^{*}$ for each of the six different landscapes.

For all landscapes, outbreak stages and grid construction methods using conditional subsample and conditional entry algorithms, even the worst grid configuration tested provided an improvement over the pairwise method, and the simulations with optimal suggested grid configurations (${\hat{\theta }}^{*}$) yielded improvements in the range of 2.9–500.0 times faster (Figs 2 and 3, S1 Fig, S2 Fig). In almost all of those simulations the CS transmission algorithm consistently performed as well as or better than the CE method (Fig 4), and for that reason we present the results for the CS algorithm here and the results for the CE algorithm can be found in the supplementary material together with the result of the optimal grid estimation method for the six landscapes (S3 Fig).

The estimated optimal grid size, ${\hat{\theta }}^{*}$, coincided with $\theta_{\mathrm{sim}}^{*}$ for half of the combinations of landscapes and outbreak stages, meaning that the optimum grid estimation method worked well. However, for the situations where it was not the optimal grid size, it was generally very close to the optimal size, the most notable exception being for the uniformly random landscape at outbreak stage 10000 for which simulations using ${\hat{\theta }}^{*}$ were 18.4% slower on average than for the optimal grid size $\theta_{\mathrm{sim}}^{*}$ (Fig 2, Table 4).

**Table 4:** Comparison between predicted optimal grid size, ${\hat{\theta }}^{*}$, and best actual grid configuration apart from ${\hat{\theta }}^{*}$.

	Relative difference in run time.			
Outbreak stage (infected nodes)	10	100	1000	10000
Random uniform	-1.2%	+1.2%	-8.5%	-18.4%
Random mod. Clustering	-6.0%	-3.0%	-3.5%	+5.3%
Random high clustering	-0.3%	+1.2%	+7.1%	+11.2%
USA	-4.9%	-0.6%	+1.4%	+3.6%
Sweden	-14.4%	+6.7%	+20.6%	+16.8%
UK	-8.6%	-7.1%	+3.2%	+15.8%

For the different outbreak size thresholds and landscapes using the conditional subsample transmission algorithm with the adaptive grid construction method, the positive values indicate that ${\hat{\theta }}^{*}$ performed best in the simulations. For these cases the numbers represent the relative increase in average run time when comparing ${\hat{\theta }}^{*}$ to the second-to-optimal grid cell size. Negative values indicate that ${\hat{\theta }}^{*}$ did not perform best in the simulations and show the relative decrease in average run time when comparing ${\hat{\theta }}^{*}$ to the value of *θ* that gave the best performance.

No difference was evident in the number of time steps required in order to reach the different stages regardless of transmission algorithm and grid configuration, supporting our claim that the methods treat the dynamics of the outbreak equally (S4 Fig, S5 Fig). Incidence curves further supporting the identical behavior for the CE and CS method to the pairwise simulations using the estimated optimal grid size ${\hat{\theta }}^{*}$ can be found as supplementary information (S6 Fig). Out of the 500 replicates for each of the 13 grid configurations, not all reached all outbreak stages. Information on the number of replicates that reached the different stages can be found in the supplementary material (S1 Table).

The regular grid construction method (Fig 1A) is only suitable for creating a grid configuration for a landscape with uniform spatial node distribution. If the node distribution is heterogeneous (as is expected for most real landscapes), the estimated optimal grid configuration will not be close to the actual optimum. This is evident from S7 Fig, S8 Fig where the results of simulating outbreaks on the landscapes with heterogeneous node distribution using the regular gridding method are shown.

**Fig 5:**
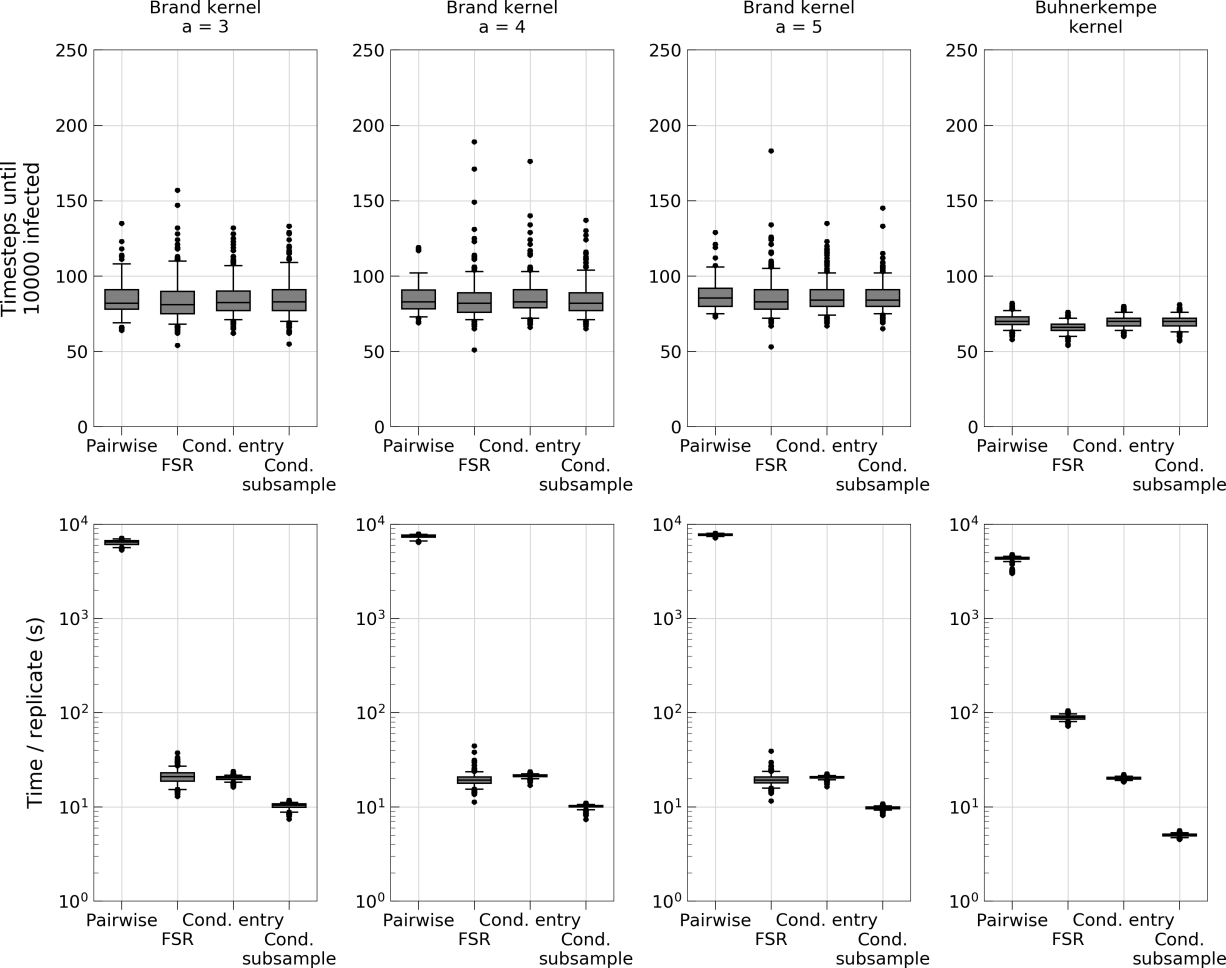
Comparison of transmission algorithms to the FSR method for outbreaks of up to 10000 infected nodes. Outliers represent the results from replicates where the epidemic died out early and consequently took considerably less time than larger outbreaks.

**Fig 6:**
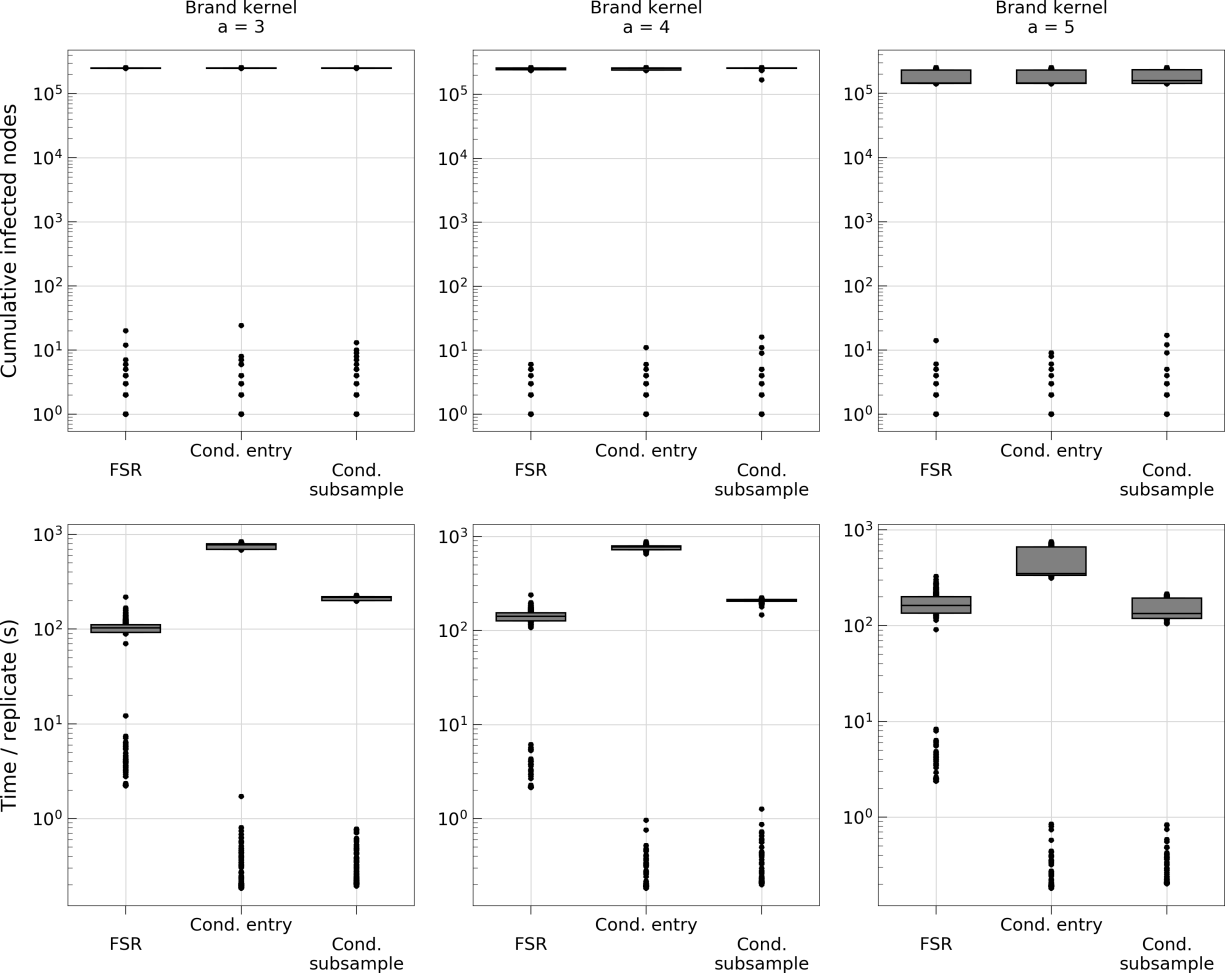
Comparison of transmission algorithms to the FSR method for simulations of outbreaks that were allowed to run their full course.

The comparison between the transmission algorithms presented in this paper with the FSR method presented in [10] showed that the CS algorithm always performed best on outbreaks with up to 10000 cumulative infected nodes (Fig 5). For simulations that ran the entire course of outbreaks the FSR method performed better for *Brand* kernels with shape parameter *a = 3* and *a = 4*, while FSR and the CS algorithm was on par for shape *a = 5* (Fig 6).

## Discussion

Stochastic disease simulation models are powerful tools for contingency planning and can be used to evaluate the efficiency of different control options [2,18,19]. However, a large number of replicates may have to be simulated to capture a representative range of possible outcomes; an issue that is amplified when different seeding conditions are included. Commonly, multiple control actions need to be considered [20,21], further inflating the number of simulations. Thus, computation time quickly becomes a limiting factor. We have introduced a novel algorithm for this purpose, denoted the conditional subsample method, and demonstrated that it yield substantial reduction of computational complexity. When compared to two other available optimization methods, it outperformed the conditional entry algorithm in almost all situations, and in most cases also the FSR algorithm.

We have in this study focused on spatially explicit simulation models, using as an application livestock disease models where farms are considered the infective unit. The introduced algorithm as well as the CE method [11] requires gridding of the spatial landscape (e.g. Fig 1), making the potential for speed-up sensitive to the grid configuration. For this purpose, we have introduced a method for estimation of the optimal number of nodes per cell. The estimation is based on the simplifying assumption that nodes are distributed randomly in a unit square. As expected, the method performs well in identifying an optimal grid size when simulating outbreaks under such ideal conditions (Fig 3A).

However, this is far too crude an assumption for most instances, as node locations are usually not randomly distributed [16]. To see how well our optimal grid size estimation method holds when this assumption is violated, we also simulated outbreaks in two computer generated landscapes with different levels of clustering, as well as three empirical landscapes of farm locations: Sweden, the UK and the USA. The analysis showed that the simulations based on the approximated optimal grid size did not perform well on these landscapes using a regular grid configuration (Fig 1A, S7 Fig, S7 Fig). However, when using adaptive grid sizes, targeting equal numbers of farms per cell, the simulations based on the estimated optimum performed very well compared to the simulations with other grid configurations (Fig 3, S1 Fig, S2 Fig). This is encouraging, suggesting that our optimal grid size estimation method can be used to decide on the grid structure for a wide range of spatial configurations. Importantly, the simulations based on the estimated optimal grid size (${\hat{\theta }}^{*}$) were substantially faster than the pairwise simulations, both during early and late stages of the outbreak (Fig 2, Fig 3, Fig 4, S1 Fig, S2 Fig). The observed speed-up when using the estimated optimal grid size ranged from a factor of 2.9 for the random high clustering landscape (161 nodes/cell, 10 infected nodes) to a factor of 500.0 for the USA (333 nodes/cell, 10000 infected nodes; Fig 2) with the CS algorithm.

The estimation of ${\hat{\theta }}^{*}$ was based on simplified representations of the landscapes. Specifically, for in order to achieve the best estimation the transmissibility and susceptibility of the nodes were based on the median or maximum farm size across the un-simplified landscape for the CE and CS algorithms, respectively. The reason for this difference is that in the CE algorithm the transmissibility used for evaluating the entry into a cell or not is based on a single infectious node which corresponds closer to the median than the maximum farm size. In the CS algorithm, however, the transmissibility used is that of the most transmissible farm in cell *a* for which maximum farm size is a better proxy than the median.

To further investigate the performance of the gridding algorithms and make comparisons to the FSR algorithm, we ran simulations with different spatial kernels, differing primarily in tail fatness [10]. We focused on the USA demography because this is the system the FSR method was introduced for. It is also the largest farm population and therefore where computational gain is the most important. Fig 5 shows that for all considered kernel shapes, the CS algorithm (using a grid configuration determined by the method described in section *Estimation of optimal grid cell size*) again outperformed the CE and pairwise methods. When comparing runtimes with the FSR method, the CS algorithm was consistently faster in terms of simulations to the first 10000 infected farms, whereas the results differed between kernel types in terms of computation time for entire outbreaks. One likely reason for this is that the FSR method works on susceptible farms and as they become fewer due to being infected and removed, the complexity of the algorithm decreases. The algorithms presented in this paper on the other hand has a complexity that grows with the number of infected farms and so, will be faster during the initial stages of the outbreak and lose relative speed as the epidemic grows. As such, there are instances where the FSR would be quicker, particularly if the simulated outbreaks are extremely large.

The FSR method introduces a slight approximation unless a suitable grid size for the image of infection can be found. No apparent difference in terms of precision is revealed in Fig 5 for the *Brand* kernels; all simulation methods provide similar estimates of number of time steps to reach either 10000 infected farms (Fig 5) or end of outbreak (Fig 6). Thus, the issue of approximation is of less concern in terms of choosing algorithm for kernels such as these. However, for the *Buhnerkempe* kernel which is particularly local, the grid requires a very fine resolution something that increases the computational burden significantly. The effect of increasing the grid resolution can be seen in the lower right panel of Fig 5 where the run time of FSR is markedly higher than for the other kernels. The grid resolution used for that simulation was high, but still too coarse to avoid introducing a slight error (upper right panel) and remedying that with an even finer grid would make the method even less competitive. It should be noted that the CS and CE algorithms are exact only beyond the discretizing into daily time steps. The Gillespie algorithm [22] can be used to model the continuous processes exactly, but to the knowledge of these authors, no optimization for this algorithm exists for the system considered here, and Brand et al. [10] showed for simulation of FMD outbreaks in the USA that the Gillespie algorithm is computationally very expensive. Beyond computation time, the discretization can be justified by the fact many process are cyclical at a finer resolution than the time scale of discretization (i.e. daily discretization and diurnal cycles), or that data is typically available at discrete time scales [10].

To explore the efficiency of the set of methods described, we have applied them to outbreaks of FMD. This is a valuable approach, because we demonstrate the applicability of the method for relevant situations. Yet, a potential objection emerges–do the results hold outside of the context we have explored? It is infeasible to consider all possible applications of the methodology; stochastic simulations of spatially explicit kernel models are used for a wide range of diseases and questions (e.g. [6,12–14]). However, the study design along with the results provide some useful insight. We simulated outbreaks in a variable assortment of spatial landscapes with varying levels of clustering and farm densities, and found that our optimization method was efficient and constantly identified a grid configuration that sped up computations substantially, ranging from 100 to 500 times faster than the pairwise estimation for outbreaks of substantial size (10000 infected nodes). Next, we used the same optimization method to identify the optimal grid configuration for different kernels and found that in this novel context, the CS method improved computation time by a factor of 600 to 800, depending on kernel. Here it also holds an edge over the FSR method when considering infections up to 10000 farms and vary in terms of computation time for extreme outbreaks (Fig 6).

These simulations suggest that the CS method is a good candidate to consider for stochastic simulations of disease outbreak. Based on Fig 6, the FSR algorithm may be faster for some applications, particularly when the simulations are expected to result in very large outbreaks. However, we are encouraged that in the instances where the CS algorithm performs slower than FSR, the difference is not vast (approximately a factor of two; Fig 6). Also, the FSR method was found to have some issues with very local kernels such as the one used in this work, so for such cases the CS or even the CE method would be more suitable.

We argue that beyond the fast computation time, one strength of the CS algorithm lies in its simplicity. Because it does not approximate the epidemic model, it requires no tweaking to avoid loss of precision. As shown in the supplement (S1 Appendix), the algorithm requires little additional code compared to the pairwise computation. In addition, the code structure is easy to combine with other processes. For instance, we have in our own ongoing work found it straightforward to combine a local transmission process, implemented with the CS algorithm, with a model for animal movements that leads to transmission over large distances [23] (see supplement S1 Appendix for example code).

Thus, we conclude that the transmission algorithms considered in this study are suitable for epidemic modeling of diseases where local area spread, modeled with a spatial kernel, is an important factor. By using the CS gridding approach, together with the introduced optimal grid cell size estimation, the computation can be sped up by several orders of magnitude. This allows for exploration of more scenarios, facilitating the use of disease simulation models for policy recommendations.

## Supporting information

S1 Table Summary of outbreak sizes for simulations. Summary of the amount of replicates that reached 10, 100, 1000 and 10000 cumulative infected nodes using the original parameter values fitted for Cumbria to the 2001 UK FMD outbreak in [15] as well as with susceptibility scaled up by a factor of 200. Initial simulation with original parameter values were only performed with the conditional subsample algorithm. Simulations with scaled parameters were performed with all three algorithms (conditional subsample algorithm, CS; conditional entry, CE; pairwise, PW). The Cumbria parameters were kept for the UK simulations but not for the other landscapes.
(XLSX)

S2 Table Summary of landscapes. Number of nodes (n) and spatial clustering measure Ripley’s L for different distances r. A value close to one indicates homogenous spatial distribution of nodes.
(XLSX)

S1 Appendix Disease simulation C++ code. (ZIP)

S2 Appendix Optimum grid size algorithm C++ code. (ZIP)

S3 Appendix Formal proof of the exactness of the CE algorithm. (PDF)

S4 Appendix Formal proof showing that the number of expected kernel calls are the same for the CE algorithm as for the CS algorithm. (PDF)

S1 Dataset An instance of the US cattle farm population as generated by FLAPS. (7Z)

S2 Dataset Randomly generated farm population with high degree of clustering. (7Z)

S3 Dataset Randomly generated farm population with moderate degree of clustering. (7Z)

S4 Dataset Randomly generated farm population with uniform spatial distribution. (7Z)

S1 FigResults from outbreak simulations with the conditional subsample transmission algorithm.Average run time in seconds for each tested grid cell size up to and including the given outbreak stages (* indicates estimated optimal grid cell size ${\hat{\theta }}^{*}$). The 5th and 95th percentiles are indicated by the ranges (main panels). Each combination of landscape and grid configuration using the CS algorithm, as well as simulations with the pairwise algorithm for comparison was simulated with 500 replicates. The landscapes were (panels A-F): random uniform, random moderate clustering, random high clustering, USA, Sweden, UK. The regular grid construction method was used for the uniform random landscape and the adaptive grid construction method was used for the other landscapes. The black line indicates a unitless relative expected efficacy of the different grid sizes as indicated by the grid optimum estimation method.(TIF)

S2 FigResults from outbreak simulations with the conditional entry algorithm and adaptive gridding.Average run time in seconds for each tested grid cell size up to and including the given outbreak stages (* indicates estimated optimal grid cell size ${\hat{\theta }}^{*}$). The 5th and 95th percentiles are indicated by the ranges (main panels). Each combination of landscape and grid configuration using the CE algorithm, as well as simulations with the pairwise algorithm for comparison was simulated with 500 replicates. The landscapes were (panels A-F): random uniform, random moderate clustering, random high clustering, USA, Sweden, UK. The regular grid construction method was used for the uniform random landscape and the adaptive grid construction method was used for the other landscapes. The black line indicates a unitless relative expected efficacy of the different grid sizes as indicated by the grid optimum estimation method.(TIF)

S3 Fig Predicted optimal gridding configurations. The estimated number of average kernel calls per cell required to simulate one time step of infection spreading from one infectious farm in each cell to all other cells on a simplified spatially uniform representation of the original landscapes. On the x-axis are the grid configurations as the square root of the total number of cells in the regular grid (κ). Results shown for the two different summary statistics median and max number of animals on each node used for calculating transmissibility and susceptibility in the estimation.
(TIF)

S4 Fig The cumulative number of infected nodes at the indicated outbreak stages for the different combinations of landscape and grid configurations for the CS algorithm as well as for the pairwise algorithm for comparison. The CS algorithm consistently gives the same number of infected nodes as the pairwise algorithm.
(TIF)

S5 Fig The cumulative number of infected nodes at the indicated outbreak stages for the different combinations of landscape and grid configurations for the CE algorithm as well as for the pairwise algorithm for comparison. The CE algorithm consistently gives the same number of infected nodes as the pairwise algorithm.
(TIF)

S6 Fig Incidence curves for simulations with the pairwise, conditional subsample and conditional entry algorithms. The x-axis shows time step and the y-axis shows the mean cumulative number of infected nodes over all the replicates, including the replicates for where the epidemic had died out which is why the curve start to decline after some time.
(TIF)

S7 Fig Results from outbreak simulations with the conditional subsample transmission algorithm and regular grid construction method. Average run time in seconds for each tested grid cell size up to and including the given outbreak stages (* indicates estimated optimal grid cell size). The 5th and 95th percentiles are indicated by the ranges (main panels). Each combination of landscape and grid configuration using the CS algorithm, as well as simulations with the pairwise algorithm for comparison using 500 replicates. Only the landscapes with heterogeneous node distribution are shown (panels A-E): random moderate clustering, random high clustering, USA, Sweden and UK. The black line indicates a unitless relative expected efficacy of the different grid sizes as indicated by the grid optimum estimation method, note the skew away from the predicted optimum.
(TIF)

S8 Fig Results from outbreak simulations with the conditional entry algorithm and regular gridding. Average run time in seconds for each tested grid cell size up to and including the given outbreak stages (* indicates estimated optimal grid cell size). The 5th and 95th percentile are indicated by the ranges (main panels). Each combination of landscape and grid configuration using the CE algorithm, as well as simulations with the pairwise algorithm for comparison was simulated with 500 replicates. Only the landscapes with heterogeneous node distribution are shown (panels A-E): random moderate clustering, random high clustering, USA, Sweden, UK. The black line indicates a unitless relative expected efficacy of the different grid sizes as indicated by the grid optimum estimation method, note the skew away from the predicted optimum.
(TIF)
