# Supplementary material for: Need for speed: An optimized gridding approach for spatially explicit disease simulations
Source: PLoS Comput Biol. 2018 Apr 6;14(4):e1006086. doi: 10.1371/journal.pcbi.1006086 (PMC5906030; doi:10.1371/journal.pcbi.1006086)
Supplement: S3 Appendix — (PDF) [file pcbi.1006086.s005.pdf]

Conditional entry method proof of exactness

The conditional entry (CE) method is built around the idea that if one can calculate the probability that the infectious node i fails to infect at least one node in another grid cell, it is possible to skip entire cells with just one random variate, saving time. The algorithm itself is described in the main text, this proof shows that the probability of a node being infected conditioned on the fact that infection has "entered" the grid cell in accordance with the description of the algorithm is equal to the original pairwise probability described by eq. 2 in the main text. We do this by proving that the sum of the probabilities for every possible outcome where node j becomes infected by i using this algorithm equals p_{ij}. For this entire proof we consider one single infectious node i and one grid cell with susceptible nodes j. Also note that while the algorithm described in the main text makes use of an over-estimated probability ν_{ib}, this over-estimation has no bearing on this proof and here we use the actual pairwise probabilities instead. Once it has been determined that infection enters the cell, each subsequent check to see if a node becomes infected must take into consideration the outcome of the earlier checks. For every node that is determined to not become infected the subsequent node must be conditioned on the fact that at least one of the remaining susceptible nodes in the cell shall be infected.

Suppose there are N susceptible nodes in the target square and let

$$p_{ij} = p_j = 1 - e^{-R_j} \tag{1}$$

denote the pairwise probability of transmission from node i to node j based on transmission rate R_j. Denote the probability of at least one of the remaining k susceptible nodes out of N becoming infected by i as

$$q_k = 1 - \prod_{j=k}^{N} (1 - p_j) = 1 - \prod_{j=k}^{N} \left(1 - \left(1 - e^{-R_j}\right)\right) = 1 - e^{-\sum_{j=k}^{N} R_j}. \tag{2}$$

When k equals 1, q_k describes the probability of infecting at least one of the susceptible nodes, the criterion for infection entering the cell.

Further, note that

$$\frac{p_k}{q_k} + \left(1 - \frac{p_k}{q_k}\right)\frac{1}{q_{k+1}} = \frac{p_k q_{k+1} + q_k - p_k}{q_k q_{k+1}} = \tag{3}$$
$$\frac{\left(1-e^{-R_k}\right)\left(1-e^{-\sum_{j=k+1}^{N} R_j}\right) + 1 - e^{-\sum_{j=k}^{N} R_j} - \left(1-e^{-R_k}\right)}{q_{n-(k-1)} q_{n-k}} =$$
$$\frac{1 - e^{-\sum_{j=k+1}^{N} R_j} - e^{-R_k} + e^{-\sum_{j=k}^{N} R_j} + 1 - e^{-\sum_{j=k}^{N} R_j} - 1 + e^{-R_k}}{q_k q_{k+1}} =$$
$$\frac{1 - e^{-\sum_{j=k+1}^{N} R_j}}{q_k q_{k+1}} = \frac{1}{q_k}.$$

The probability of the first node becoming infected in the algorithm is simply

$$q_1 \frac{p_1}{q_1} = p_1 \tag{4}$$

The probability of node 2 being infected is calculated as the sum of the probabilities that (conditional on the event that at least one of N nodes becomes infected):

- node 1 and node 2 infected
- node 1 not infected and node 2 infected,

i.e.

$$q_1 \frac{p_1}{q_1} p_2 + q_1 \left(1 - \frac{p_1}{q_1}\right) \frac{p_2}{q_2}. \tag{5}$$

For simplicity, we introduce the notation

$$F_j = \frac{p_j}{q_j} \text{ and} \tag{6}$$
$$\bar{F}_j = 1 - F_j = 1 - \frac{p_j}{q_j}.$$

From eq. (3) and (6) we get

$$F_j + \bar{F}_j \frac{1}{q_{j+1}} = \frac{1}{q_j}. \tag{7}$$

We use the notation introduced in (6) and write eq. (5) as

$$q_1 F_1 p_2 + q_1 \bar{F}_1 \frac{p_2}{q_2} = \tag{8}$$
$$q_1 p_2 \left(\underbrace{F_1 + \bar{F}_1 \frac{1}{q_2}}_{=\frac{1}{q_1}\text{(eq. 7)}}\right) = q_1 p_2 \frac{1}{q_1} = p_2,$$

The probability of the third node being infected is analogously calculated as the sum of the probabilities (conditional on at least one of N nodes gets infected)

- node 1 and node 3 infected
- node 1 not infected, node 2 and node 3 infected
- node 1 and node 2 not infected, node 3 infected.

i.e.

$$\begin{gathered}
q_1 \tfrac{p_1}{q_1} p_3 + q_1 \left(1 - \tfrac{p_1}{q_1}\right) \tfrac{p_2}{q_2} p_3 + q_1 \left(1 - \tfrac{p_1}{q_1}\right) \left(1 - \tfrac{p_2}{q_2}\right) \tfrac{p_3}{q_3} = \\
q_1 p_3 \left[\tfrac{p_1}{q_1} + \left(1 - \tfrac{p_1}{q_1}\right) \tfrac{p_2}{q_2} + \left(1 - \tfrac{p_1}{q_1}\right) \left(1 - \tfrac{p_2}{q_2}\right) \tfrac{1}{q_3}\right] = \\
q_1 p_3 \left[F_1 + \bar{F}_1 F_2 + \bar{F}_1 \bar{F}_2 \tfrac{1}{q_3}\right].
\end{gathered} \tag{9}$$

Let

$$\begin{aligned}
\Sigma_1 &= F_1 \\
\Sigma_2 &= F_1 + \bar{F}_1 F_2 \\
&\vdots \\
\Sigma_i &= F_1 + \bar{F}_1 F_2 + \ldots + \bar{F}_1 \cdots \bar{F}_{i-1} F_i
\end{aligned} \tag{10}$$

With the notation above, we can write the probabilities of node 2 and node 3 as

$$\begin{gathered}
q_1 p_2 \left(F_1 + \bar{F}_1 \frac{1}{q_2}\right) = q_1 p_2 \left(\Sigma_1 + \bar{F}_1 \frac{1}{q_2}\right) \text{ and} \\
q_1 p_3 \left(F_1 + \bar{F}_1 F_2 + \bar{F}_1 \bar{F}_2 \frac{1}{q_3}\right) = q_1 p_3 \left(\Sigma_2 + \bar{F}_1 \bar{F}_2 \frac{1}{q_3}\right)
\end{gathered} \tag{11}$$

For arbitrary node $k \leq N$, the probability according to the algorithm is written as

$$\begin{gathered}
q_1 p_k \left(F_1 + \bar{F}_1 F_2 + \ldots + \bar{F}_1 \bar{F}_2 \cdots F_{k-1} + \bar{F}_1 \bar{F}_2 \cdots \bar{F}_{k-1} \frac{1}{q_k}\right) = \\
q_1 p_k \left(\Sigma_{k-1} + \bar{F}_1 \bar{F}_2 \cdots \bar{F}_{k-1} \frac{1}{q_k}\right).
\end{gathered} \tag{12}$$

Note that

$$\begin{gathered}
\Sigma_1 + \bar{F}_1 \frac{1}{q_2} = \frac{1}{q_1} \text{ and} \\
\Sigma_2 + \bar{F}_1 \bar{F}_2 \frac{1}{q_3} = F_1 + \bar{F}_1 F_2 + \bar{F}_1 \bar{F}_2 \frac{1}{q_3} = \\
F_1 + \bar{F}_1 \left(F_2 + \bar{F}_2 \frac{1}{q_3}\right) = \Sigma_1 + \bar{F}_1 \frac{1}{q_2} = \frac{1}{q_1}
\end{gathered} \tag{13}$$

Thus

$$\Sigma_k + \bar{F}_1 \cdot \ldots \cdot \bar{F}_k \frac{1}{q_{k+1}} = \frac{1}{q_1} \tag{14}$$

holds for node $k \in \{1, 2\}$. Suppose eq. (14) holds for an arbitrary $k < N$, then we have for node $k + 1$,

$$\Sigma_{k+1} + \bar{F}_1 \cdots \bar{F}_{k+1} \frac{1}{q_{k+2}} = \tag{15}$$

$$F_1 + \bar{F}_1 F_2 + \ldots + \bar{F}_1 \cdots \bar{F}_k F_{k+1} + \bar{F}_1 \cdots \bar{F}_{k+1} \frac{1}{q_{k+2}} = \tag{16}$$

$$\Sigma_k + \bar{F}_1 \cdots \bar{F}_k F_{k+1} + \bar{F}_1 \cdots \bar{F}_{k+1} \frac{1}{q_{k+2}} = \tag{17}$$

$$\Sigma_k + \bar{F}_1 \cdots \bar{F}_k \left(F_{k+1} + \bar{F}_{k+1} \frac{1}{q_{k+2}} \right) = \tag{18}$$

$$\Sigma_k + \bar{F}_1 \cdots \bar{F}_k \frac{1}{q_{k+1}} = \frac{1}{q_1}. \tag{19}$$

Thus,

$$q_N p_k \left(F_1 + \bar{F}_1 F_2 + \ldots + \bar{F}_1 \bar{F}_2 \cdots F_{k-1} + \bar{F}_1 \bar{F}_2 \cdots \bar{F}_{k-1} \frac{1}{q_k} \right) = p_k \quad \forall k \leq N, \tag{20}$$

that is, the probability of node k being infected in the conditional entry algorithm is equal to p_k as given by eq. 1.
