# Supplementary material for: Need for speed: An optimized gridding approach for spatially explicit disease simulations
Source: PLoS Comput Biol. 2018 Apr 6;14(4):e1006086. doi: 10.1371/journal.pcbi.1006086 (PMC5906030; doi:10.1371/journal.pcbi.1006086)
Supplement: S4 Appendix — (PDF) [file pcbi.1006086.s006.pdf]

S4 Appendix
Expected number of kernel calls, conditional entry method.

Let i be an infectious node in cell a, and $b \neq a$ be a cell containing n susceptible nodes. Let $\nu_{i,b}$ be an overestimated probability of infection describing the probability that i infects a hypothetical node in b defined so that $\nu_{i,b}$ is the probability of transmission from the infectious node i to the node with the highest probability of becoming infected within the destination square (for the definition of $\nu_{i,b}$ used in this study, see eq. (7) in section 4.2.2 in the main paper).

Further, let $N_{i,b}$ denote the expected number of calls to the kernel function required for testing if infection spreads from i to any of the n susceptible nodes within b

$$N_{i,b} = \omega_{i,b}((n - m_n)\nu_{i,b} + 1), \tag{1}$$

where $\omega_{i,b}$ is the probability of "entering" the cell (the probability that at least one hypothetical node with probability of infection $\nu_{i,b}$ becomes infected)

$$\omega_{i,b} = (1 - (1 - \nu_{i,b})^n) \tag{2}$$

and m_n is the expected number of hypothetical nodes that need to be tested until one of them becomes infected. The explanation of (1) is that upon entering a cell the conditional algorithm iterates through all susceptible nodes within the cell and checks for actual infection. This is done in two steps for each node, first by testing if the node would have been infected given $\nu_{i,b}$ *conditional on* that at least one of susceptible "hypothetical" nodes that remain to be iterated over becomes infected:

$$prob_{hypothetical} = \frac{\nu_{i,b}}{(1 - \nu_{i,b})^{n_{rem.}}} \tag{3}$$

If this is the case, the second step is to test for actual infection by calculating the *true* probability for the current node to become infected. Therefore, the kernel has only to be evaluated for the actual inter-node distance for the nodes that are not discarded in the first step. After the first such occurrence, the condition that at least one "hypothetical" node becomes infected is fulfilled and the remaining iterations use

$$prob_{hypothetical} = \nu_{i,b}. \tag{4}$$

Therefore, no kernel function calls need to be performed up until the first hypothetical infection, at which point one call always occurs (the added one in (1)).

After that, each remaining node to iterate over will require a kernel function call with probability $\nu_{i,b}$. This expected number of remaining nodes after the first hypothetical infection is described by $n - m_n$ in (1) where m_n is given by

$$m_n = \sum_{j=1}^{n} \underbrace{\frac{j\nu_{i,b}}{1-(1-\nu_{i,b})^{n+1-j}} \begin{cases} 1 & j=1 \\ \prod_{k=1}^{k=j-1}\left(1-\frac{\nu_{i,b}}{1-(1-\nu_{i,b})^{n+1-k}}\right) & j>1 \end{cases}}_{\mu_j}. \tag{5}$$

Now, denote element j of the sum in (5) with μ_j.

Lemma 0.1. *Let* $0 < \nu_{i,b} < 1$, $n > 0$ *and*

$$\mu_j = \frac{j\nu_{i,b}}{1-(1-\nu_{i,b})^{n+1-j}} \begin{cases} 1 & j=1 \\ \prod_{k=1}^{k=j-1}\left(1-\frac{\nu_{i,b}}{1-(1-\nu_{i,b})^{n+1-k}}\right) & j>1 \end{cases}, \tag{6}$$

then

$$\mu_j = j\,(1-\nu_{i,b})^{j-1}\,\mu_1.$$

Proof. Let $q = 1 - \nu_{i,b}$, this gives

$$\mu_1 = \frac{\nu_{i,b}}{1-(1-\nu_{i,b})^n} = \frac{1-q}{1-q^n}. \tag{7}$$

For $j > 1$

$$\begin{aligned} \mu_j &= \frac{j\nu_{i,b}}{1-(1-\nu_{i,b})^{n+1-j}} \prod_{k=1}^{k=j-1}(1-\frac{\nu_{i,b}}{1-(1-\nu_{i,b})^{n+1-k}}) = \\ &= \frac{j\,(1-q)}{1-q^{n+1-j}} \prod_{k=1}^{k=j-1}\left(1-\frac{1-q}{1-q^{n+1-k}}\right) = \\ &= \frac{j\,(1-q)}{1-q^{n+1-j}} \prod_{k=1}^{k=j-1}\left(\frac{q-q^{n+1-k}}{1-q^{n+1-k}}\right) \end{aligned} \tag{8}$$

and

$$\begin{aligned} \mu_{j+1} &= \frac{(j+1)\,(1-q)}{1-q^{n+1-(j+1)}} \prod_{k=1}^{k=(j+1)-1}\left(\frac{q-q^{n+1-k}}{1-q^{n+1-k}}\right) = \\ &= \frac{(j+1)\,(1-q)\left(q-q^{n+1-j}\right)}{\left(1-q^{n+1-(j+1)}\right)\left(1-q^{n+1-j}\right)} \prod_{k=1}^{k=j-1}\left(\frac{q-q^{n+1-k}}{1-q^{n+1-k}}\right) = \\ &= \frac{(j+1)\,(1-q)\,q\left(1-q^{n-j}\right)}{\left(1-q^{n-j}\right)\left(1-q^{n+1-j}\right)} \prod_{k=1}^{k=j-1}\left(\frac{q-q^{n+1-k}}{1-q^{n+1-k}}\right) = \\ &= \frac{(j+1)\,jq\,(1-q)}{j\left(1-q^{n+1-j}\right)} \prod_{k=1}^{k=j-1}\left(\frac{q-q^{n+1-k}}{1-q^{n+1-k}}\right) = \frac{(j+1)\,q}{j}\mu_j. \end{aligned} \tag{9}$$

From (8),we get for $j = 2$,

$$\mu_2 = \frac{2\nu_{i,b}}{1-(1-\nu_{i,b})^{n-1}}(1-\frac{\nu_{i,b}}{1-(1-\nu_{i,b})^{n+1-1}}) =$$
$$= \frac{2(1-q)}{1-q^{n-1}}\left(1-\frac{1-q}{1-q^n}\right) = \frac{2(1-q)}{1-q^{n-1}}\left(\frac{q-q^n}{1-q^n}\right) =$$
$$= \frac{2q(1-q)}{1-q^{n-1}}\left(\frac{1-q^{n-1}}{1-q^n}\right) = \frac{2q(1-q)}{1-q^n} = 2q\mu_1 = \frac{2q}{1}\mu_1.$$

Note that (9) implies, for $j = 2$

$$\mu_3 = \frac{3q}{2}\mu_2 = \frac{3q}{2}\frac{2q}{1}\mu_1 = 3q^2\mu_1,$$

thus

$$\mu_j = jq^{j-1}\mu_1 \tag{10}$$

for $j = 2$ and $j = 3$.

Now, suppose that

$$\mu_j = jq^{j-1}\mu_1 \quad j > 3. \tag{11}$$

This, together with (9) implies

$$\mu_{j+1} = \frac{(j+1)\,q}{j}\mu_j = \frac{(j+1)\,q}{j}\left(jq^{j-1}\mu_1\right) = (j+1)\,q^j\mu_1$$
$$= (j+1)\,(1-\nu_{i,b})^j\,\mu_1.$$

□

Lemma 0.2. *Let m_n be defined as in (5) and let $q = 1-\nu_{i,b}$, then*

$$m_n = \frac{1-q^n\,(1+n\,(1-q))}{(1-q)\,(1-q^n)},\ n > 1.$$

Proof. From (5) we get

$$m_n = \sum_{j=1}^{n}\frac{j\nu_{i,b}}{1-(1-\nu_{i,b})^{n+1-j}}\prod_{k=1}^{k=j-1}(1-\frac{\nu_{i,b}}{1-(1-\nu_{i,b})^{n+1-k}}) = \sum_{j=1}^{n}\mu_j.$$

Lemma 0.1 and $q = 1 - \nu_{i,b}$ implies

$$\begin{aligned}\sum_{j=1}^{n} \mu_j =& \mu_1 \sum_{j=1}^{n} j q^{j-1} = \frac{1-q}{1-q^n}\left(1 + 2q + 3q^2 + \ldots + nq^{n-1}\right) = \\ =& \frac{\left(1 + 2q + 3q^2 + \ldots + nq^{n-1} - q - 2q^2 - 3q^3 \ldots - nq^n\right)}{1-q^n} = \\ =& \frac{\left(1 + q + q^2 + \ldots + q^{n-1} - nq^n\right)}{1-q^n} = \\ =& \frac{(1-q)\left(1 + q + q^2 + \ldots + q^{n-1} - nq^n\right)}{(1-q)(1-q^n)} = \\ =& \frac{1 + q + q^2 + \ldots + q^{n-1} - nq^n - q - q^2 - q^3 - \ldots - q^n + nq^{n+1}}{(1-q)(1-q^n)} = \\ =& \frac{1 - (n+1) q^n + nq^{n+1}}{(1-q)(1-q^n)} = \frac{1 - q^n (n + 1 - nq)}{(1-q)(1-q^n)} = \\ =& \frac{1 - q^n (1 + n(1-q))}{(1-q)(1-q^n)}.\end{aligned}$$

□

We can now simplify the number of expected kernel function calls in eq. (1).

Theorem 0.3. *The expected number of kernel calls, as defined in(1), can be written as*

$$N_{i,b} = n\nu_{i,b}.$$

Proof. Let $N_{i,b}$ be defined as in eq. (1) and let $q = 1 - \nu_{i,b}$, then

$$N_{i,b} = ((n - m_n)\nu_{i,b} + 1)(1 - (1 - \nu_{i,b})^n) = ((n - m_n)(1-q) + 1)(1 - q^n).$$

Lemma 0.2 implies

$$\begin{aligned}N_{i,b} =& \left(\left(n - \frac{1 - q^n (1 + n(1-q))}{(1-q)(1-q^n)}\right)(1-q) + 1\right)(1-q^n) = \\ =& \left(\left(n(1-q) - \frac{1 - q^n (1 + n(1-q))}{(1-q^n)}\right) + 1\right)(1-q^n) = \\ =& n(1-q)(1-q^n) - (1 - q^n (1 + n(1-q))) + (1-q^n) = \\ =& n(1-q)(1-q^n) - \left(1 - q^n - nq^n + nq^{n+1}\right) + 1 - q^n = \\ =& n - nq - nq^n + nq^{n+1} - 1 + q^n + nq^n - nq^{n+1} + 1 - q^n = \\ =& n(1-q) = n\nu_{i,b}.\end{aligned}$$

□
