# Supplementary figures and images for: Need for speed: An optimized gridding approach for spatially explicit disease simulations
Source: PLoS Comput Biol. 2018 Apr 6;14(4):e1006086. doi: 10.1371/journal.pcbi.1006086 (PMC5906030; doi:10.1371/journal.pcbi.1006086)

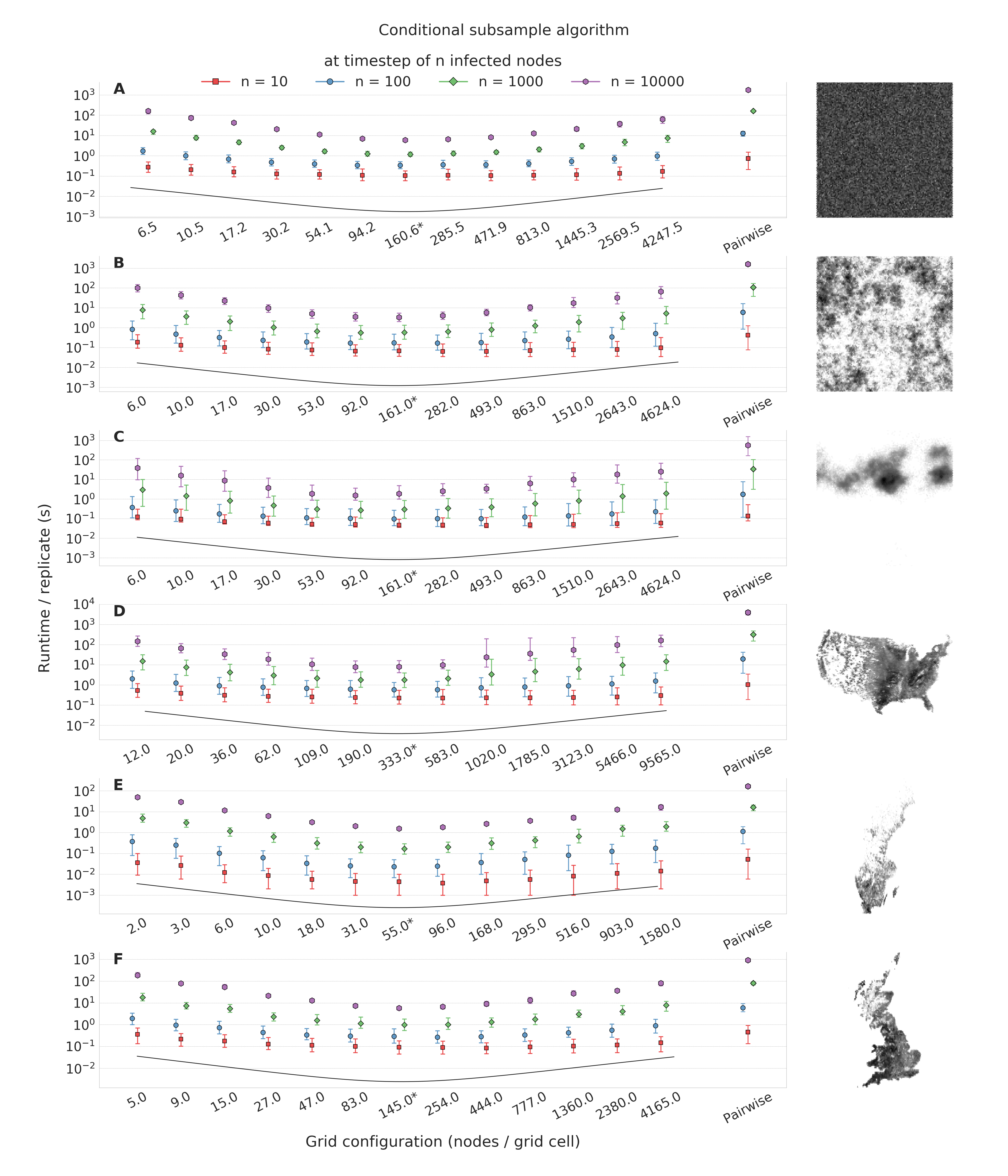

Supplement: S1 Fig — Average run time in seconds for each tested grid cell size up to and including the given outbreak stages (* indicates estimated optimal grid cell size θ^*). The 5th and 95th percentiles are indicated by the ranges (main panels). Each combination of landscape and grid configuration using the CS algorithm, as well as simulations with the pairwise algorithm for comparison was simulated with 500 replicates. The landscapes were (panels A-F): random uniform, random moderate clustering, random high clustering, USA, Sweden, UK. The regular grid construction method was used for the uniform random landscape and the adaptive grid construction method was used for the other landscapes. The black line indicates a unitless relative expected efficacy of the different grid sizes as indicated by the grid optimum estimation method. (TIF) [file pcbi.1006086.s011.tif]

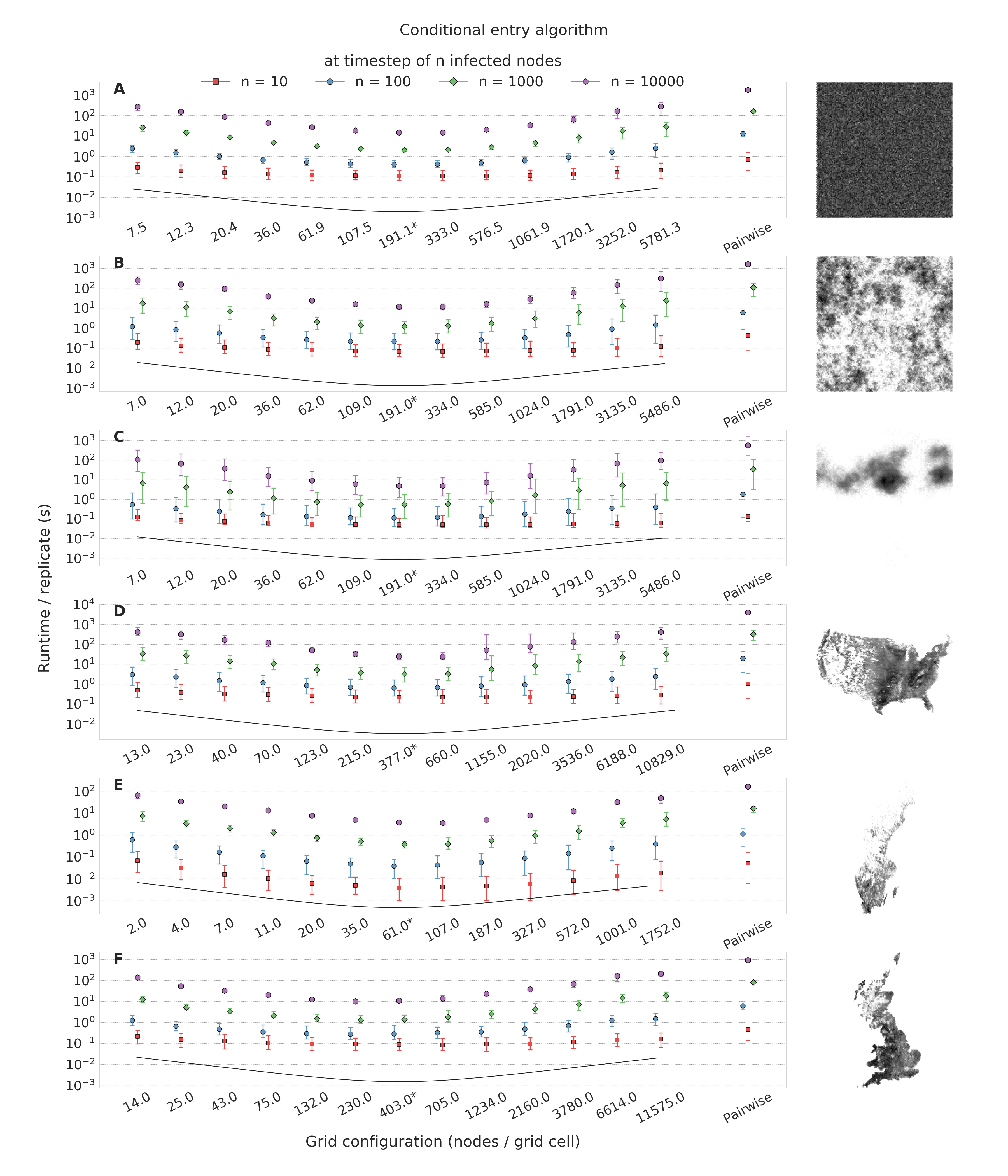

Supplement: S2 Fig — Average run time in seconds for each tested grid cell size up to and including the given outbreak stages (* indicates estimated optimal grid cell size θ^*). The 5th and 95th percentiles are indicated by the ranges (main panels). Each combination of landscape and grid configuration using the CE algorithm, as well as simulations with the pairwise algorithm for comparison was simulated with 500 replicates. The landscapes were (panels A-F): random uniform, random moderate clustering, random high clustering, USA, Sweden, UK. The regular grid construction method was used for the uniform random landscape and the adaptive grid construction method was used for the other landscapes. The black line indicates a unitless relative expected efficacy of the different grid sizes as indicated by the grid optimum estimation method. (TIF) [file pcbi.1006086.s012.tif]

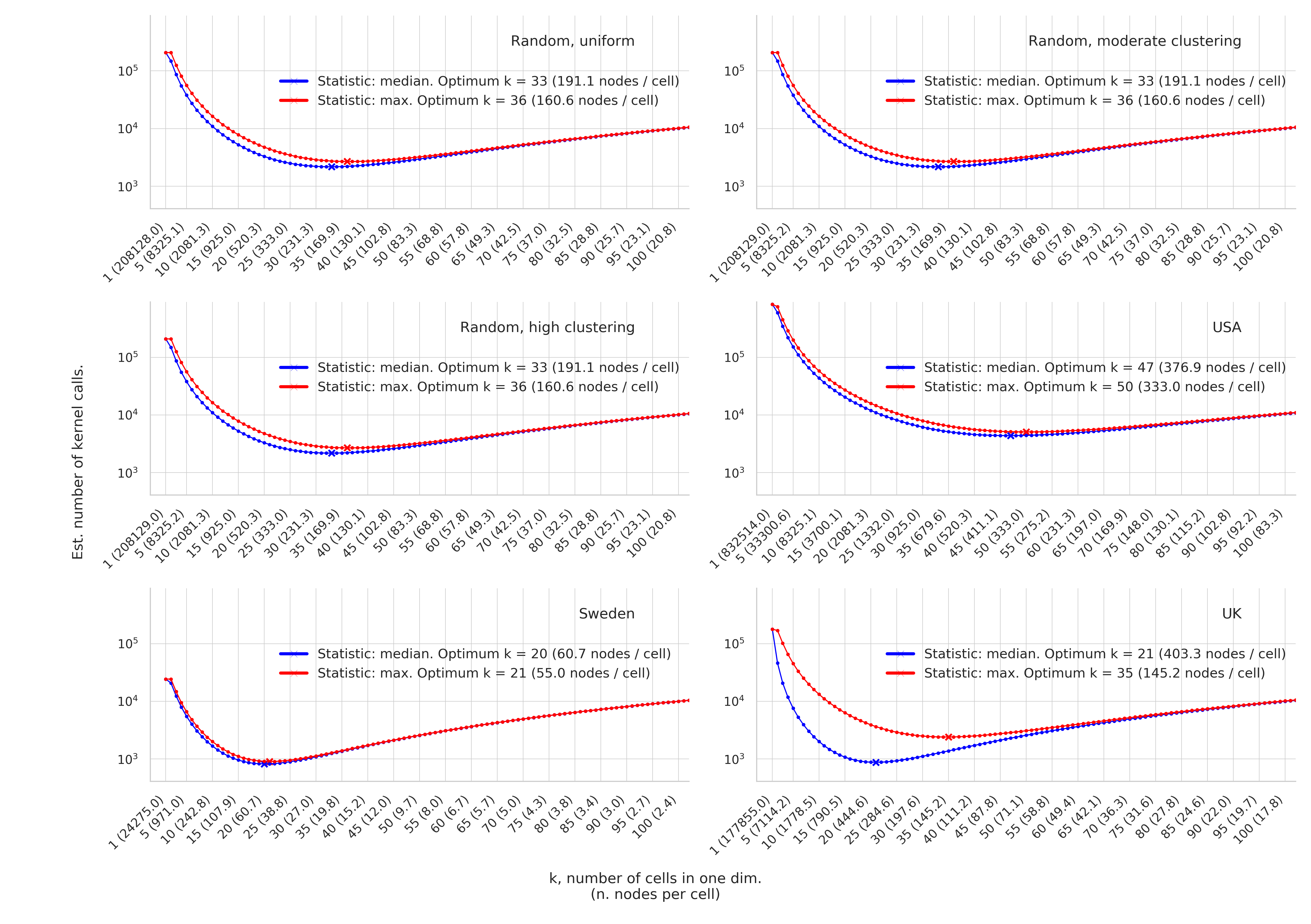

Supplement: S3 Fig — The estimated number of average kernel calls per cell required to simulate one time step of infection spreading from one infectious farm in each cell to all other cells on a simplified spatially uniform representation of the original landscapes. On the x-axis are the grid configurations as the square root of the total number of cells in the regular grid (κ). Results shown for the two different summary statistics median and max number of animals on each node used for calculating transmissibility and susceptibility in the estimation. (TIF) [file pcbi.1006086.s013.tif]

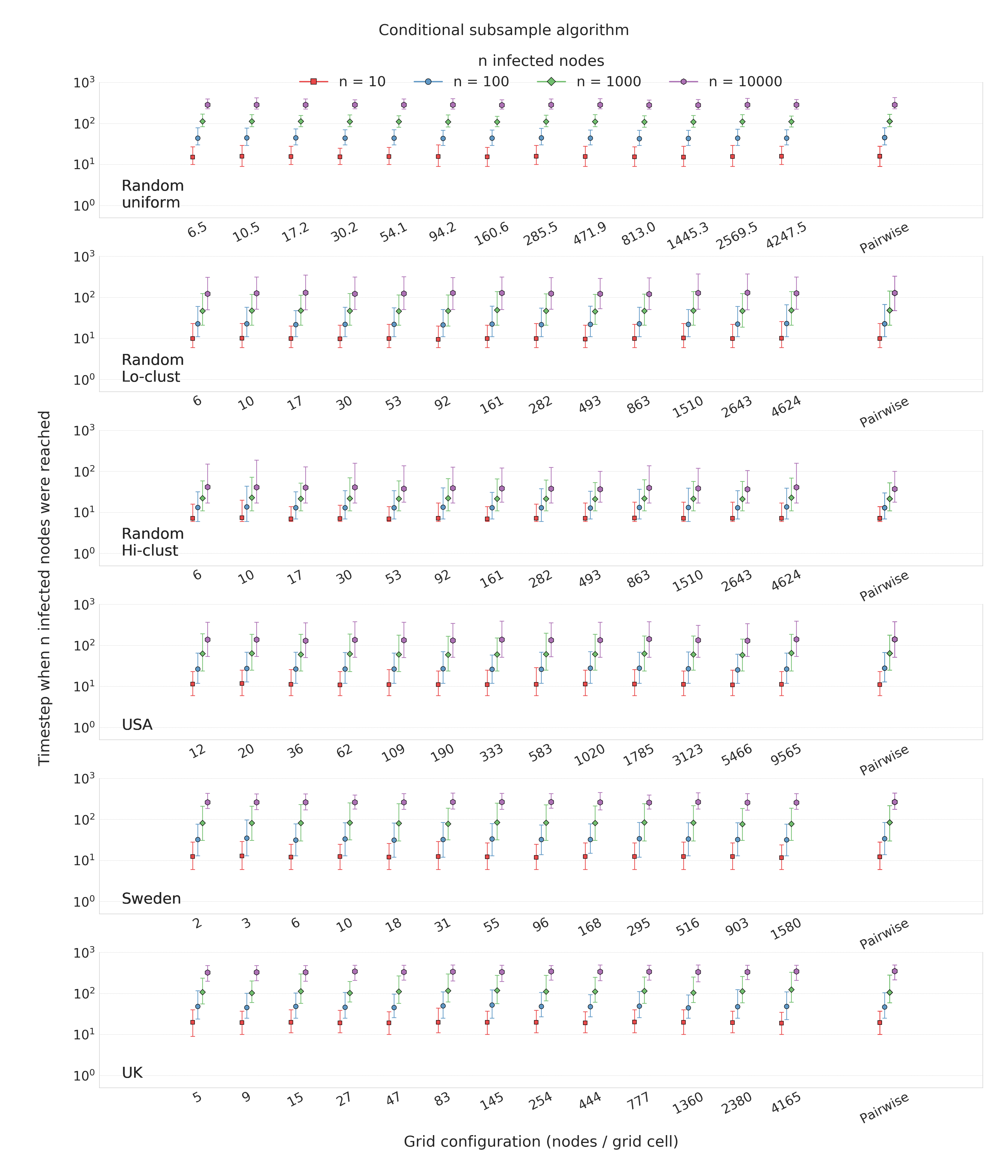

Supplement: S4 Fig — The CS algorithm consistently gives the same number of infected nodes as the pairwise algorithm. (TIF) [file pcbi.1006086.s014.tif]

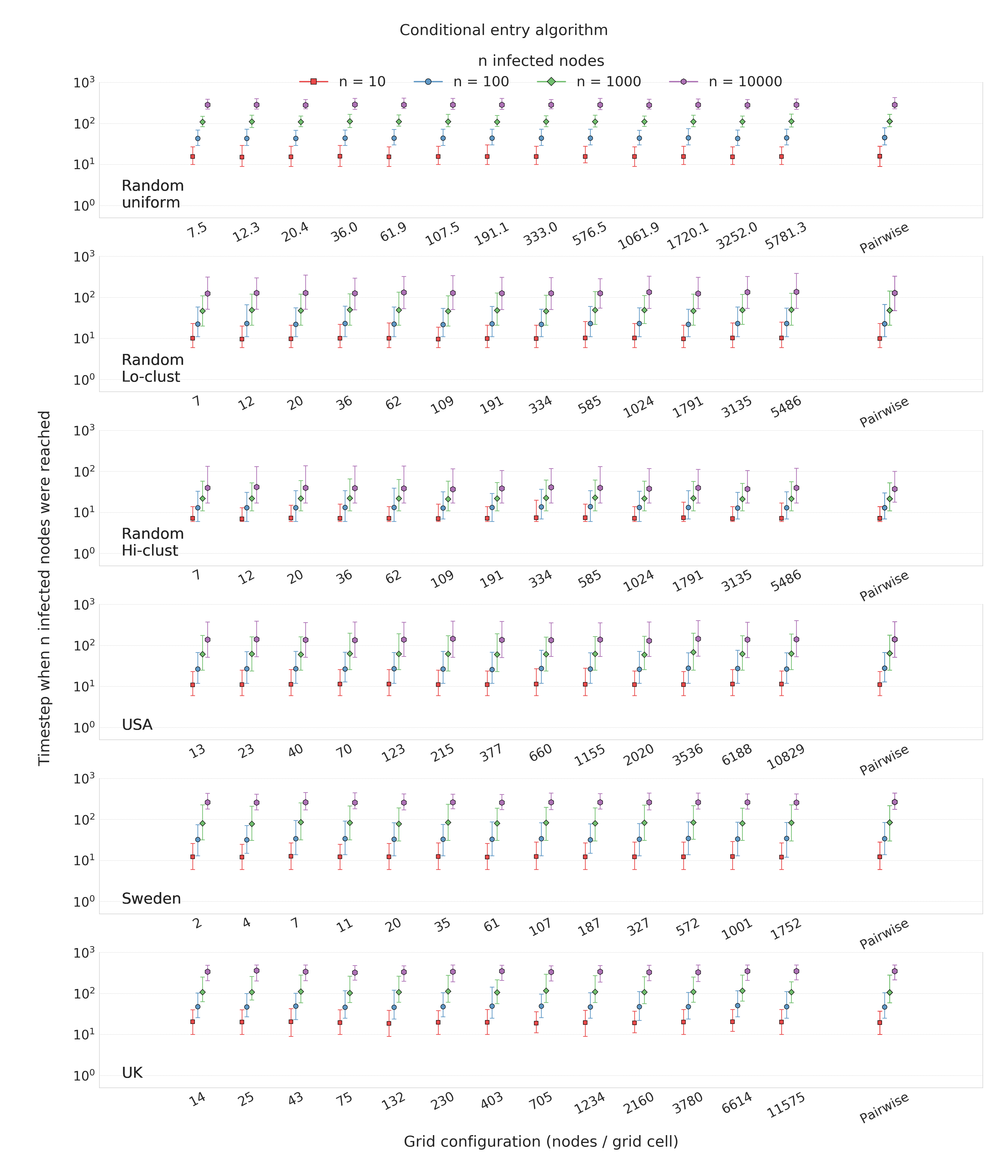

Supplement: S5 Fig — The CE algorithm consistently gives the same number of infected nodes as the pairwise algorithm. (TIF) [file pcbi.1006086.s015.tif]

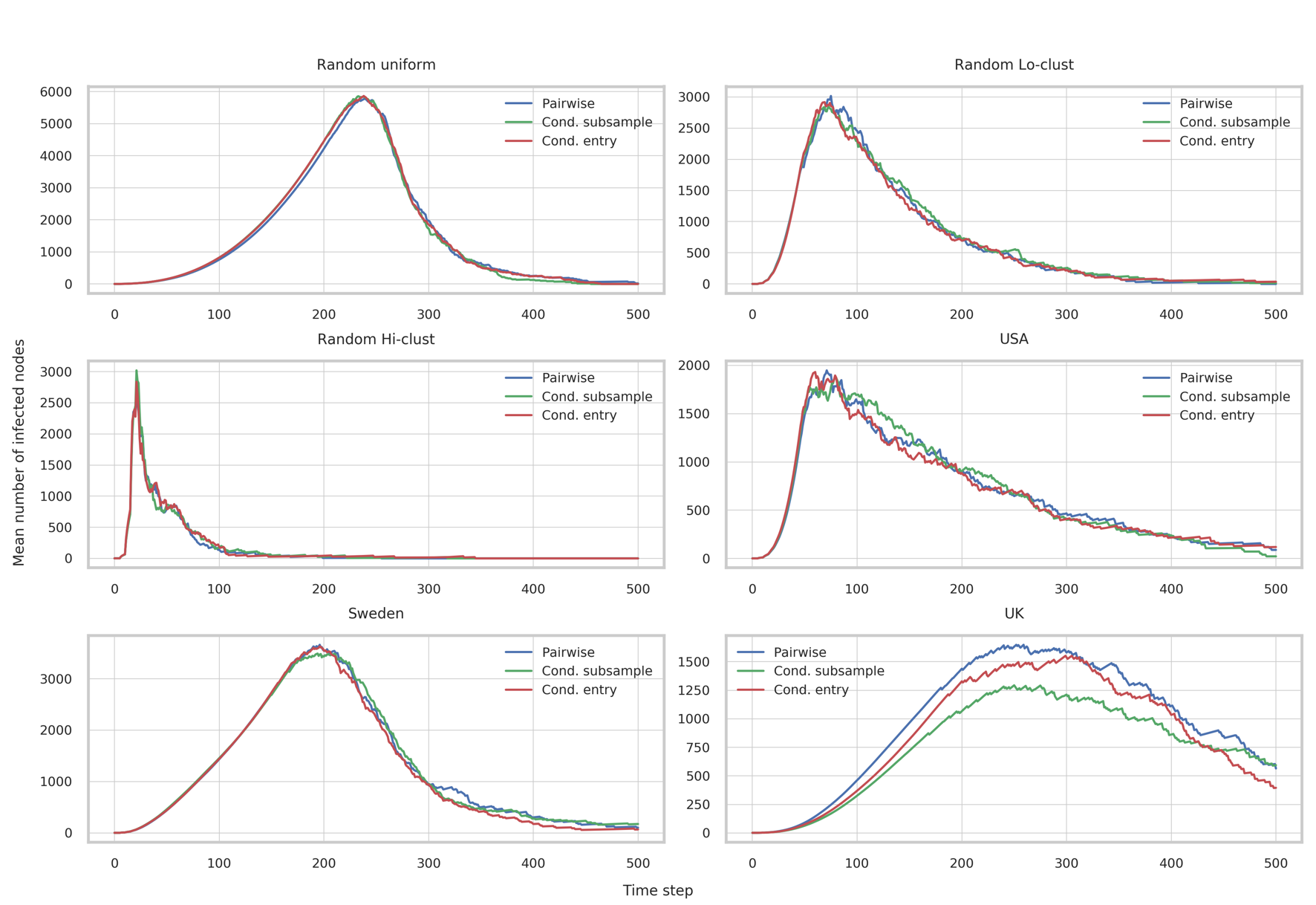

Supplement: S6 Fig — The x-axis shows time step and the y-axis shows the mean cumulative number of infected nodes over all the replicates, including the replicates for where the epidemic had died out which is why the curve start to decline after some time. (TIF) [file pcbi.1006086.s016.tif]

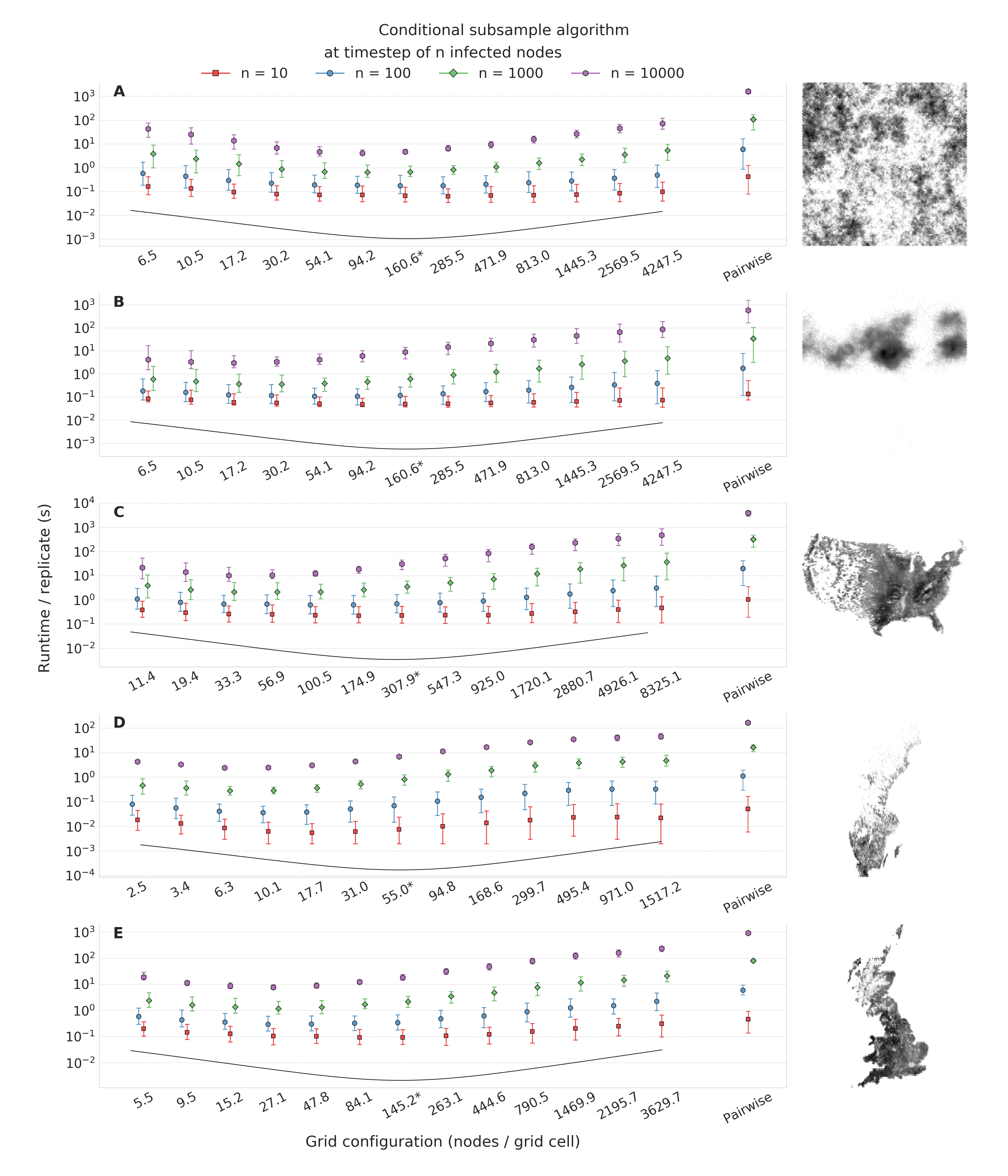

Supplement: S7 Fig — Average run time in seconds for each tested grid cell size up to and including the given outbreak stages (* indicates estimated optimal grid cell size). The 5th and 95th percentiles are indicated by the ranges (main panels). Each combination of landscape and grid configuration using the CS algorithm, as well as simulations with the pairwise algorithm for comparison using 500 replicates. Only the landscapes with heterogeneous node distribution are shown (panels A-E): random moderate clustering, random high clustering, USA, Sweden and UK. The black line indicates a unitless relative expected efficacy of the different grid sizes as indicated by the grid optimum estimation method, note the skew away from the predicted optimum. (TIF) [file pcbi.1006086.s017.tif]

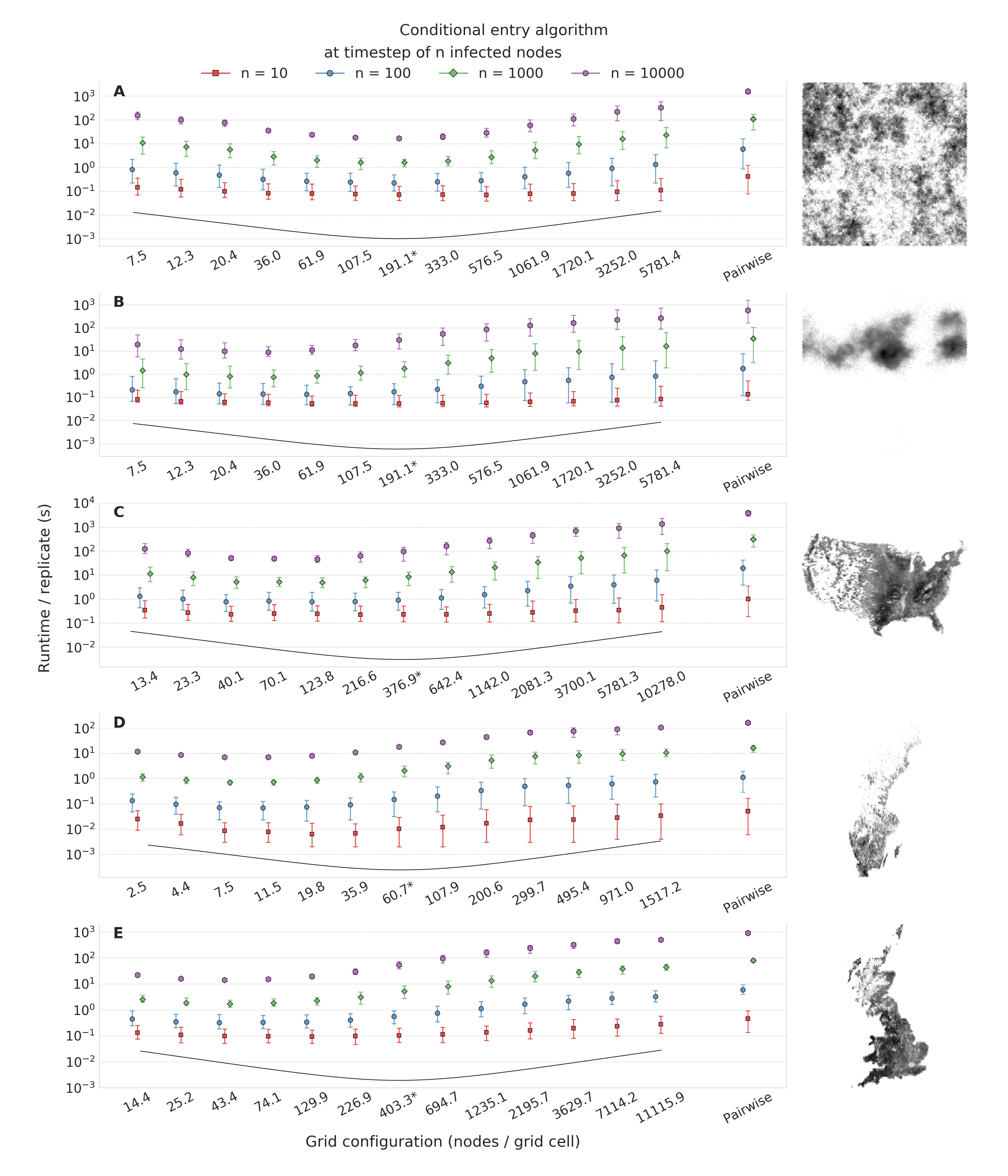

Supplement: S8 Fig — Average run time in seconds for each tested grid cell size up to and including the given outbreak stages (* indicates estimated optimal grid cell size). The 5th and 95th percentile are indicated by the ranges (main panels). Each combination of landscape and grid configuration using the CE algorithm, as well as simulations with the pairwise algorithm for comparison was simulated with 500 replicates. Only the landscapes with heterogeneous node distribution are shown (panels A-E): random moderate clustering, random high clustering, USA, Sweden, UK. The black line indicates a unitless relative expected efficacy of the different grid sizes as indicated by the grid optimum estimation method, note the skew away from the predicted optimum. (TIF) [file pcbi.1006086.s018.tif]
